# ATP6AP2 in osteoblasts couples cortical bone matrix remodeling to osteocyte maturation via MMP14 activation in mice

**DOI:** 10.1038/s42003-026-10615-z

**Published:** 2026-07-11

**Authors:** Lei Xiong, Hao-Han Guo, Jin-Xiu Pan, Xiao Ren, Daehoon Lee, Lin Mei, Wen-Cheng Xiong

**Affiliations:** Department of Neurosciences, School of Medicine, Case Western Reserve University, Cleveland, OH USA

**Keywords:** Proteolysis, Bone remodelling

## Abstract

Cortical bone is a dense, lamellar structure containing osteoblast (OB)-lineage–derived osteocytes embedded within a mineralized matrix. How osteocytes differentiate and organize within lamellar bone remains incompletely understood. Here, we show that ATP6AP2 in the OB lineage is essential for osteocyte maturation and cortical bone development. Male mice with OB-lineage–specific deletion of *Atp6ap2* (*Atp6ap2*^*OCN-Cre*^) exhibit increased cortical bone mass composed largely of irregular woven bone, with impaired osteocyte maturation and survival, and abnormal osteocyte distribution. Mechanistic analyses identify matrix metalloproteinase-14 (MMP14) as a key downstream effector of ATP6AP2. ATP6AP2 interacts with MMP14 and promotes its surface localization largely in immature osteocytes, and expression of MMP14 in *Atp6ap2*-deficient OB-lineage cells diminishes cortical bone defects. These results reveal previously unrecognized roles for ATP6AP2 in regulating cortical osteocyte development and establish an ATP6AP2–MMP14 signaling axis that controls the woven-to-lamellar bone transition and osteocyte differentiation and distribution.

## Abstract (web summary)

An ATP6AP2–MMP14 signalling axis links extracellular matrix remodeling to osteocyte maturation and woven-to-lamellar bone transition during cortical bone development in mice.

## Introduction

Cortical bone is the dense, compact bone forming the outer layer of long bones. Modeling and remodeling cortical bones are essential for maintaining bone strength and repairing microdamage from everyday mechanical stress^1,2^. Dysregulation of this process can lead to disorders such as osteoporosis, osteopetrosis, or increased fracture risk^1,2^. Therefore, understanding the mechanisms underlying cortical bone remodeling is of great interest, as it may provide insights into disease pathogenesis and identify potential therapeutic targets.

Cortical bone development follows distinct temporal and spatial patterns compared with trabecular bone. It begins with the formation of woven bone, which is subsequently replaced by lamellar bone deposited on the woven scaffold^3,4^. During this process, osteoblasts (OBs) initially lay down collagen fibers in an irregular orientation, so called the woven bone, which is remodeled over time into a mechanically stronger lamellar structure, characterized by parallel collagen fibers arranged in perpendicular planes across adjacent lamellae and containing flattened, aligned osteocytes^3–5^. Despite these cellular processes, the precise mechanisms underlying the formation of compact cortical bone remain incompletely understood.

Osteocytes, the most abundant cells in mature cortical bone, are highly interconnected via cellular processes and are active in protein synthesis and modification^6–9^. They regulate OB and osteoclast (OC) activity to control bone remodeling, maintain systemic phosphate and calcium homeostasis, sense mechanical loading, and secrete factors affecting other organs, including the heart, liver, and kidney^6,7,9–13^. Osteocytes arise from mesenchymal stem cells through OB differentiation, passing through several transitional stages: pre-OB, OB, embedding OB, osteoid-osteocyte, mineralizing osteocyte, and mature osteocyte^14,15^. Traditionally, OB embedding was considered passive, with cells “buried alive” under matrix produced by neighboring OBs^14,16^. Recent evidence, however, suggests embedding is an active, invasive process requiring matrix degradation to form lacunae and canaliculi^17,18^. Despite these insights, the precise molecular and cellular mechanisms driving OB embedding, osteocyte maturation, and network formation remain poorly understood.

ATP6AP2 (ATPase H^+^ transporting accessory protein 2) plays critical roles in multiple signaling pathways and functions across diverse organs^19–23^. ATP6AP2 was initially identified as PRR (pro-renin receptor), because of its ability to bind to pro-renin and renin, thereby facilitating the conversion of angiotensinogen to angiotensin I^22^. Subsequent studies demonstrated that ATP6AP2 also serves as an accessory subunit of the vacuolar H⁺-ATPase (V-ATPase), a multi-subunit proton pump complex, where it regulates V-ATPase assembly and activity^24,25^. In addition, ATP6AP2 has been recognized as an essential regulator of Wnt/β-catenin signaling^26,27^. It interacts with Wnt receptors, LRP6 (Low-Density Lipoprotein Receptor-related Protein 6) and frizzled, in HEK293 cells, *Xenopus laevis tadpoles*, and *Drosophila*^26,27^. Suppression of ATP6AP2 in HEK293 cells or in *Drosophila* reduces Wnt canonical (β-catenin) and non-canonical (PCP, planar cell polarity) signaling^26,27^.

Consistent with these findings, we recently reported that ATP6AP2 interacts with LRP6 in osteoblastic cells, where it promotes LRP6 cell surface localization and sustains both basal and Wnt-induced β-catenin signaling, thereby regulating OB differentiation and trabecular bone formation^28^. Notably, ATP6AP2-dependent Wnt/β-catenin signaling is not restricted to OBs but is also present in osteoclasts^29^. Previous studies have primarily focused on the role of this regulatory mechanism in trabecular bone, whereas its function in cortical bone remains insufficiently characterized^28^. These observations raise an important unresolved question: does ATP6AP2 in osteoblasts contribute to cortical bone remodeling and homeostasis, and if so, through what mechanisms?

Here, we show that mice with OB-lineage–specific knockout of *Atp6ap2* (*Atp6ap2*^*OCN-Cre*^) exhibit increased cortical bone formation and mass. However, this increase is abnormal, characterized by a woven bone–like matrix, minimal lamellar bone, clusters of enlarged immature osteocytes, and impaired osteocyte maturation and network formation. Mass spectrometry of plasma membrane proteins in *Atp6ap2*-KO OB-lineage cells identified MMP14, whose cell surface localization and activity were lost both in vitro and in vivo. Mechanistic studies indicate that ATP6AP2 interacts with MMP14 to promote its surface targeting. Expression of MMP14 in *Atp6ap2*-deficient osteoblast-lineage cells, achieved using a Cre-dependent lentiviral targeting strategy to enable cell-type–specific gene restoration in cortical OB-lineage cells, restored collagen-degradative activity and partially rescued osteocyte distribution, cellular processes, and maturation in *Atp6ap2*^*OCN-Cre*^ mice. Together, these findings identify ATP6AP2 as a novel regulator of MMP14, promoting the transition from woven to lamellar bone and supporting osteocyte development.

## Results

### Increased unhealthy cortical bone-mass in *Atp6ap2*^*OCN-Cre*^ mice

**Fig. 1:**
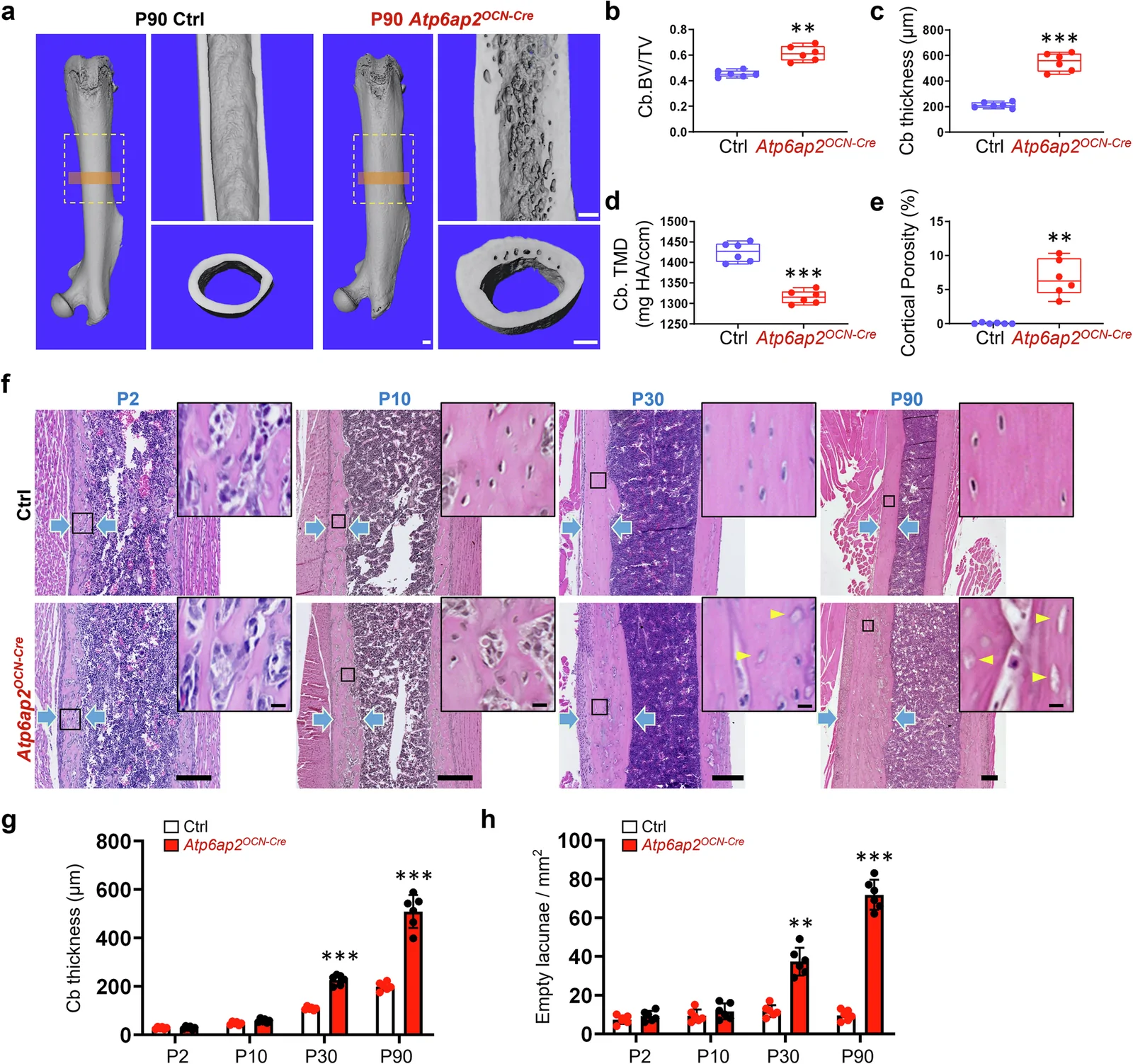
Increased unhealthy cortical bone-mass in *Atp6ap2*^*OCN-Cre*^ mice. **a** Representative μCT 3D images of femurs from P90 male ctrl and *Atp6ap2*^*OCN-Cre*^ mice littermates. Bar, 500 μm. **b**–**e** Quantification analyses of cortical bone (Cb) volumes over total volumes (BV/TV), Cb thickness, Cb tissue mineral density (TMD) and Cb porosity by direct model of μCT analysis. **f** Representative images of H&E stained femur sections from male ctrl and *Atp6ap2*^*OCN-Cre*^ mice at ages of P2, P10, P30 and P90. Yellow arrowhead indicted the empty lacunae. Bar, 200 μm in the main images and 15 μm in the boxed enlarged views. **g**, **h** Quantification analyses of Cb thickness and empty lacunae (without osteocytes) in (**f**). Data in (**b**–**e**) are shown as box plots with individual data points. The center line indicates the median, the box indicates the interquartile range, and whiskers indicate minimum to maximum values (*n* = 6 animals per genotype). Data in (**g**, **h**) are shown as mean ± SD (*n* = 6 male mice of each genotype). Statistical analysis was performed using one-way ANOVA followed by Tukey’s post hoc test. **P < 0.01. ***P < 0.001.

To investigate ATP6AP2’s function in bone, we generated male OB-selective *Atp6ap2* conditional knockout mice, *Atp6ap2*^*OCN-Cre*^, by crossing *Atp6ap2*^*flox/X*^ mice with osteocalcin (OCN)-Cre mice, whose Cre expression is directed by the human bone gamma carboxyglutamate (BGLAP) promoter/enhancer, and expresses Cre largely in the OB-lineage cells, including bone marrow stromal cells (BMSCs), OBs, and osteocytes^20,30–32^. The knockout effect of ATP6AP2 was validated by Q-PCR in multiple primary cultured cells (Fig. S1). We then examined femur bone structures in the *Atp6ap2*^*OCN-Cre*^ mice at age of P90 by micro-computed tomographic (μCT) analysis. Unexpectedly, elevation in the cortical bone volumes/total volumes (Cb, BV/TV) and cortical thickness were detected in the mutant mice, as compared with that of control littermates (*OCN-Cre*) (Fig. 1a–c). However, despite increased cortical thickness, the cortical bone exhibited markedly lower cortical bone tissue mineral density (TMD) and elevated porosity (Fig. 1d, e), indicating compromised bone quality rather than a healthy gain of bone mass.

We further examined the femur cortical bone phenotypes of the mice at ages of P2, P10, P30, and P90 by H&E staining analysis. An obvious increase in cortical bone thickness as well as more empty lacunae (without osteocytes) were detected at P30 and P90 mutant mice, but little to no changes were observed at P2 and P10 mutants, as compared with those of control mice (*OCN-Cre*) (Fig. 1f–h), supporting the view for an unhealthy cortical bone’s presence during cortical bone modeling/remodeling.

**Fig. 2:**
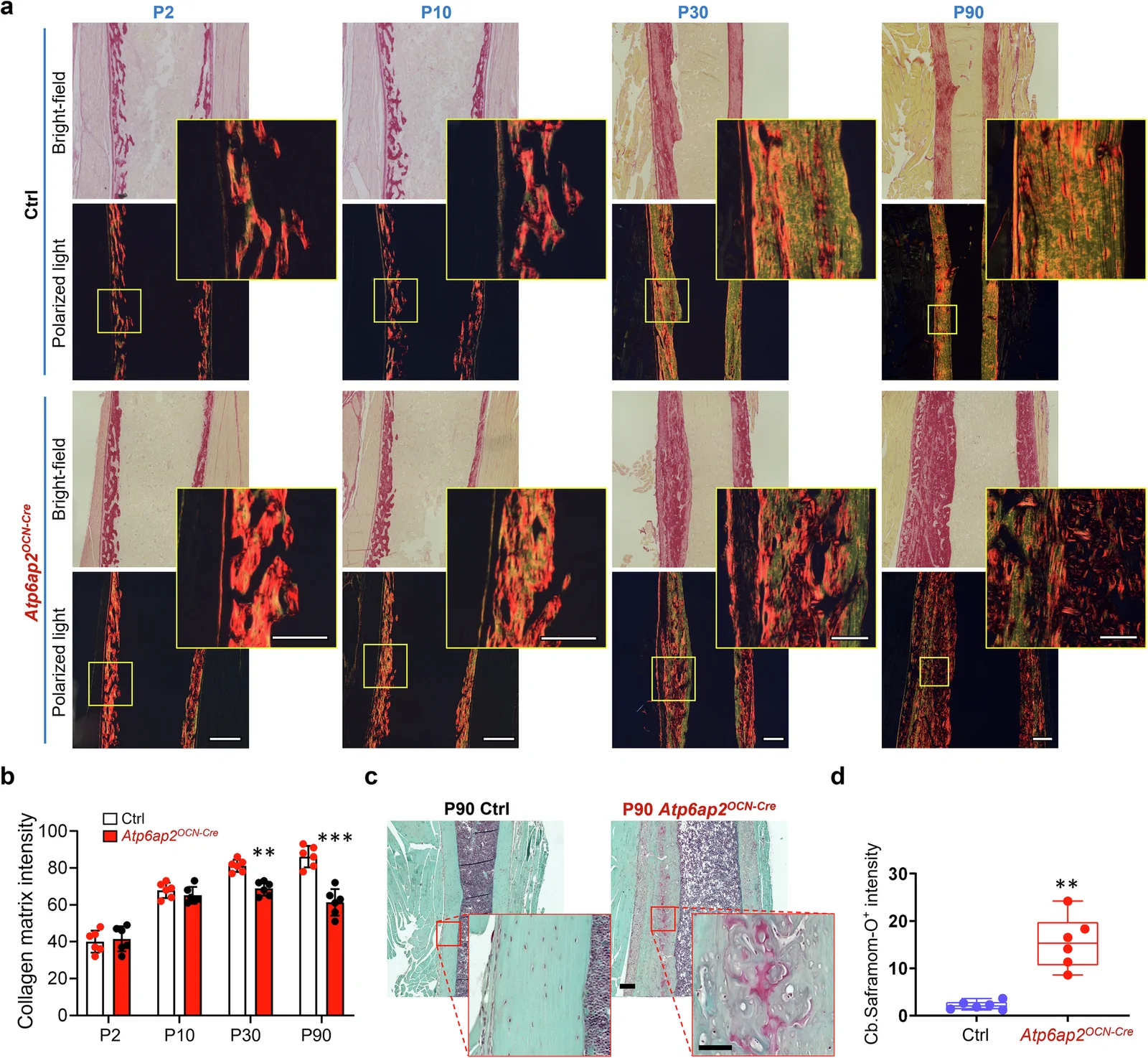
Impaired woven bone-to-lamellar bone transition in *Atp6ap2*^*OCN-Cre*^ mice. **a** Representative images of Sirius red stained cortical bone (diaphysis region) from male ctrl and *Atp6ap2*^*OCN-Cre*^ mice at ages of P2, P10 and P30 and P90. Under polarized light, the collagen fiber bundles are fine and regularly arranged in parallel (yellow, orange and green color) in P30 and P90 ctrl mice, shows the features of lamellar bone. However, the collagen fibers appeared disorganized and thicker (deep red color) in P30 and P90 mutant mice, shows the features of woven bone. From P30 age, most woven cortical bone of the ctrl mice had been replaced with lamellar bone, while the cortical bone of *Atp6ap2*^*OCN-Cre*^ mice still had a large amount of woven bone. Bar, 150 μm in the main images and 50 μm in the boxed enlarged views. **b** Quantification of collagen matrix intensity in (**a**). **c** Representative images of Safranin-O stained femur from P90 male ctrl and *Atp6ap2*^*OCN-Cre*^ mice. Bar, 300 μm in the main images and 80 μm in the boxed enlarged views. **d** Quantification of cartilage intensity in the cortical bone region (diaphysis). Data in (**b**) is presented as mean ± SD (*n* = 6 animals per genotype). Data in (**d**) is shown as box plots with individual data points. The center line indicates the median, the box indicates the interquartile range, and whiskers indicate minimum to maximum values (*n* = 6 animals per genotype). **P < 0.01. ***P < 0.001 as determined by one-way ANOVA followed by Tukey’s post hoc test or unpaired two-tailed *t* test.

We speculate that the abnormal cortical bone-mass in the mutant mice might be woven bones, which are supposed to be replaced with highly organized lamellar bone during cortical bone development^5,33^. We further tested this view by use of Picro-sirius red staining of bone sections and imaging by both bright field and polarized light to view cortical bone matrix^34,35^. Indeed, we detected many disorganized and thick collagen fibers, features of “woven” bone, in P2 and P10 control and mutant cortical bone sections (Fig. 2a, in deep red color). At ages of P30 and older (e.g., P90), the collagen fiber bundles appeared to be thinner and regularly arranged in parallel, exhibiting features of lamellar bone, in control mice (Fig. 2a, yellow, orange and green color). However, in *Atp6ap2*^*OCN-Cre*^ mice, a large number of woven bones remained at the ages of both P30 and P90 (Fig. 2a, b). These results thus support the view for an impairment in woven bone to lamellar bone transition in the mutant mice.

We also tested above view by use of Safranin-O staining (which is often used to detect cartilage matrix)^35^. Interestingly, as shown in Fig. 2c, Safranin-O positive (+) signals were detected in the metaphysis region of the cortical bones in the P90 mutant mice (Fig. 2c, d), suggesting abnormal distribution of the Safranin-O^+^ matrix, and providing additional evidence for the presence of abnormal or immature cortical bone matrix in the mutant mice.

Taken together, these results support the view for a deficit in woven bone to lamellar or mineralized bone transition in the mutant mice, which may underline the increase of abnormal cortical bone formation.

### Disorganized osteocyte distribution and increased cell proliferation, bone formation and cell death in *Atp6ap2*^*OCN-Cre*^ cortical bone

**Fig. 3:**
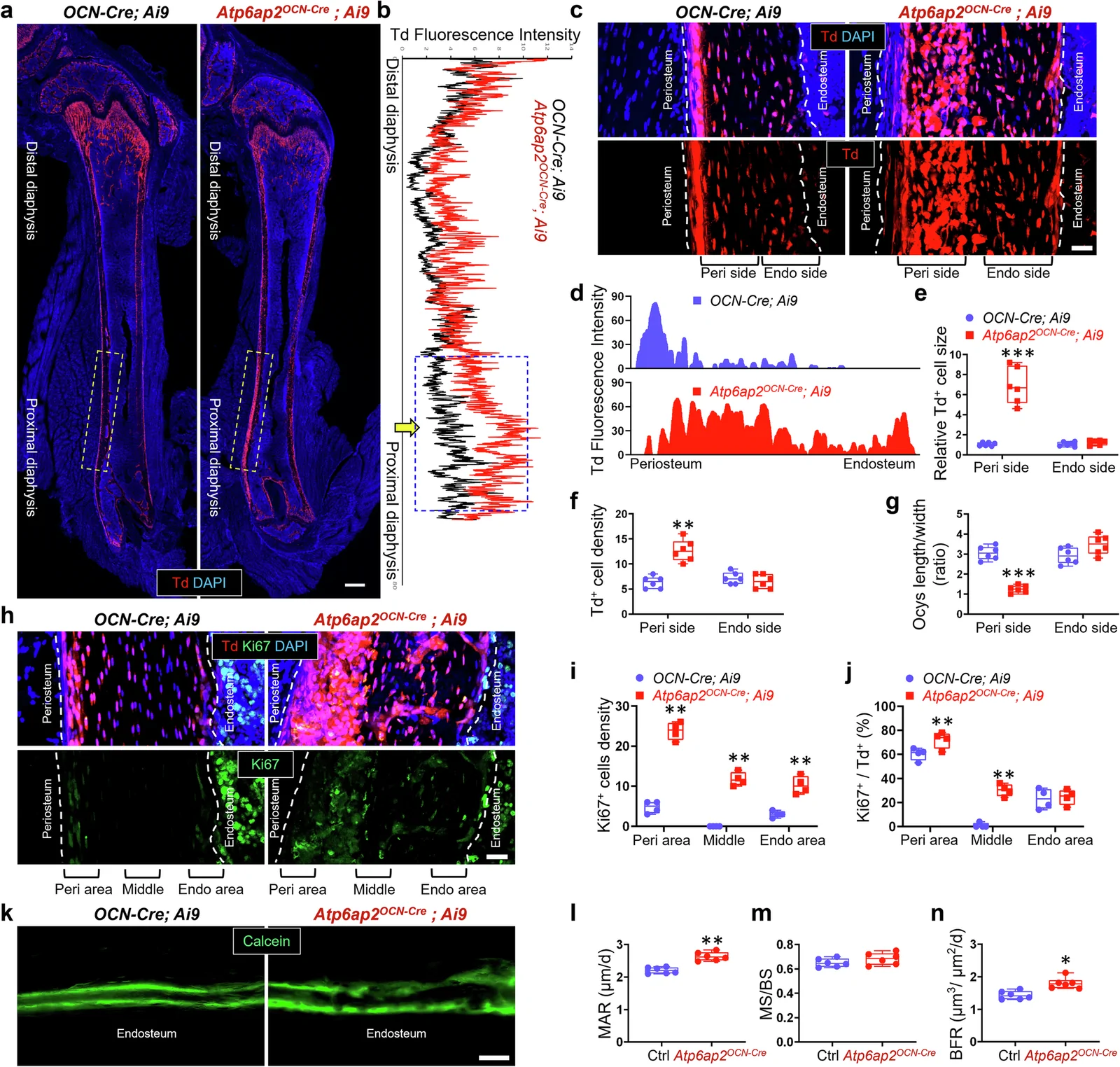
Disorganized osteocyte distribution and increased cell proliferation and bone formation in the cortical bone of *Atp6ap2*^*OCN-Cre*^ mice. **a**, **b** Td-Tomato expression on frozen femur sections from P30 *OCN-Cre; Ai9* and *Atp6ap2*^*OCN-Cre*^*; Ai9* mice. Bar, 300 μm. The Td-Tomato fluorescence intensity was shown in B. The dotted box indicated the marked increase of Td^+^ cells at the proximal diaphysis side in posterior face region. **c** Td-Tomato expression in the cortical bone region of femur sections from P30 *OCN-Cre; Ai9* and *Atp6ap2*^*OCN-Cre*^*; Ai9* mice. Bar, 50 μm. **d**–**g** Quantifications of the TdTomato red fluorescence intensity (**d**), the tdTomato^+^ cell size (**e**), cell density (**f**) and cell spherical shape (**g**). **h**–**j** Immunofluorescence of femur cortical bone in P30 *OCN-Cre; Ai9* and *Atp6ap2*^*OCN-Cre*^*; Ai9* mice by using Ki67 (cell proliferation marker) antibody. Bar, 20 μm. Quantification analysis was shown in (**i**, **j**). **k**–**n** Representative images of histologic sections showing calcein labeling of endocortical bone in femur mid-diaphysis of male Ctrl and *Atp6ap2*^*OCN-Cre*^ mice at age of P30. Bar, 50 μm. MAR (mineral apposition rate), MS (mineral surface)/BS (bone surface), and BFR (bone formation rate) are presented in **l**–**n**. Data in (**e**–**g**), (**i**), (**j**) and (**l**–**n**) are shown as box plots with individual data points. The center line indicates the median, the box indicates the interquartile range, and whiskers indicate minimum to maximum values (*n* = 4–6 animals per genotype). *P* values obtained by unpaired two-tailed *t* test. *P < 0.05. **P < 0.01. ***P < 0.001.

To understand how *Atp6ap2*-KO in OB-lineage cells increases cortical woven bones, we crossed Ai9 mice, which express tdTomato in a Cre dependent manner, with *OCN-Cre* and *Atp6ap2*^*OCN-Cre*^ mice to generate control (*OCN-Cre; Ai9*) and *Atp6ap2*^*OCN-Cre*^*; Ai9* mice, respectively, which allow us to visualize tdTomato^+^ bone cells in control and mutant bones. In line with the results of elevated cortical bone thickness, the tdTomato^+^ (or OCN-Cre^+^) cells were much more abundant in *Atp6ap2*^*OCN-Cre*^*; Ai9* cortical bones (at age of P30), as compared with those of littermate control mice (*OCN-Cre; Ai9*) (Fig. 3a, b). Notice that the increased tdTomato^+^ OBs or osteocytes in the mutant cortical bones appeared to be un-evenly distributed, with a marked increase at the proximal diaphysis side in posterior face region (Fig. 3a, b). Higher power images further showed that while the tdTomato^+^ osteocytes were evenly distributed in the cortical bone of ctrl mice, in the mutant mice, these tdTomato^+^ cells were largely disorganized, with enlarged cell size and altered cell shape, clustered in their distribution, and localized largely in the region close to the periosteum (Fig. 3c–g). These results not only support the view of increased cortical bone thickness in the mutant mice, but also demonstrate severe disruptions in osteocyte distribution and morphology in *Atp6ap2*^*OCN-Cre*^ mice (at age of P30).

We further examined cell proliferation and cell death in *Atp6ap2*^*OCN-Cre*^ cortical bones by immunofluorescence labeling analyses. As shown in Fig. 3h–j, the Ki67^+^ proliferative cells were largely distributed in the bone marrow and the periosteum or endosteum area of the control cortical bone, however, in the mutant mice, in addition to bone marrow, a dramatic increase in Ki67^+^ cells were detected in the cortical bone region (Fig. 3h, i). Note that more Ki67^+^ cells were co-labeled with tdTomato in the periosteum side, implying that these enlarged cells have more proliferative capacity (Fig. 3j). Additionally, the cleaved caspase-3^+^ apoptotic cells were also detected in the cortical bone of the mutant mice (Fig. S2a, b). These cleaved caspase-3^+^ cells were co-labeled with tdTomato, largely distributed in the middle or on the periosteum side of the cortical bone, implying that cell death occurs mainly in osteocytes (Fig. S2). We then examined bone formation in the cortical bones of the control and mutant mice by injecting fluorochrome-labeled calcein green into P30 mice twice at a 5-days interval. The mineral apposition rate (MAR), mineral surface/bone surface (MS/BS), and bone formation rate (BFR) of endocortical bone in mid-diaphysis were analyzed in non-decalcified femur bone sections from the injected mice. The MAR, MS/BS and BFR were increased in *Atp6ap2*^*OCN-Cre*^ mice, as compared with those of control mice (Fig. 3k–n). These results suggest increased abnormal cell proliferation, cell death and bone formation in *Atp6ap2*^*OCN-Cre*^ cortical bones, which may underlie the elevated cortical bone thickness and empty lacunae in the mutant mice.

### Defective osteocyte maturation and processes in *Atp6ap2*^*OCN-Cre*^ cortical bones

**Fig. 4:**
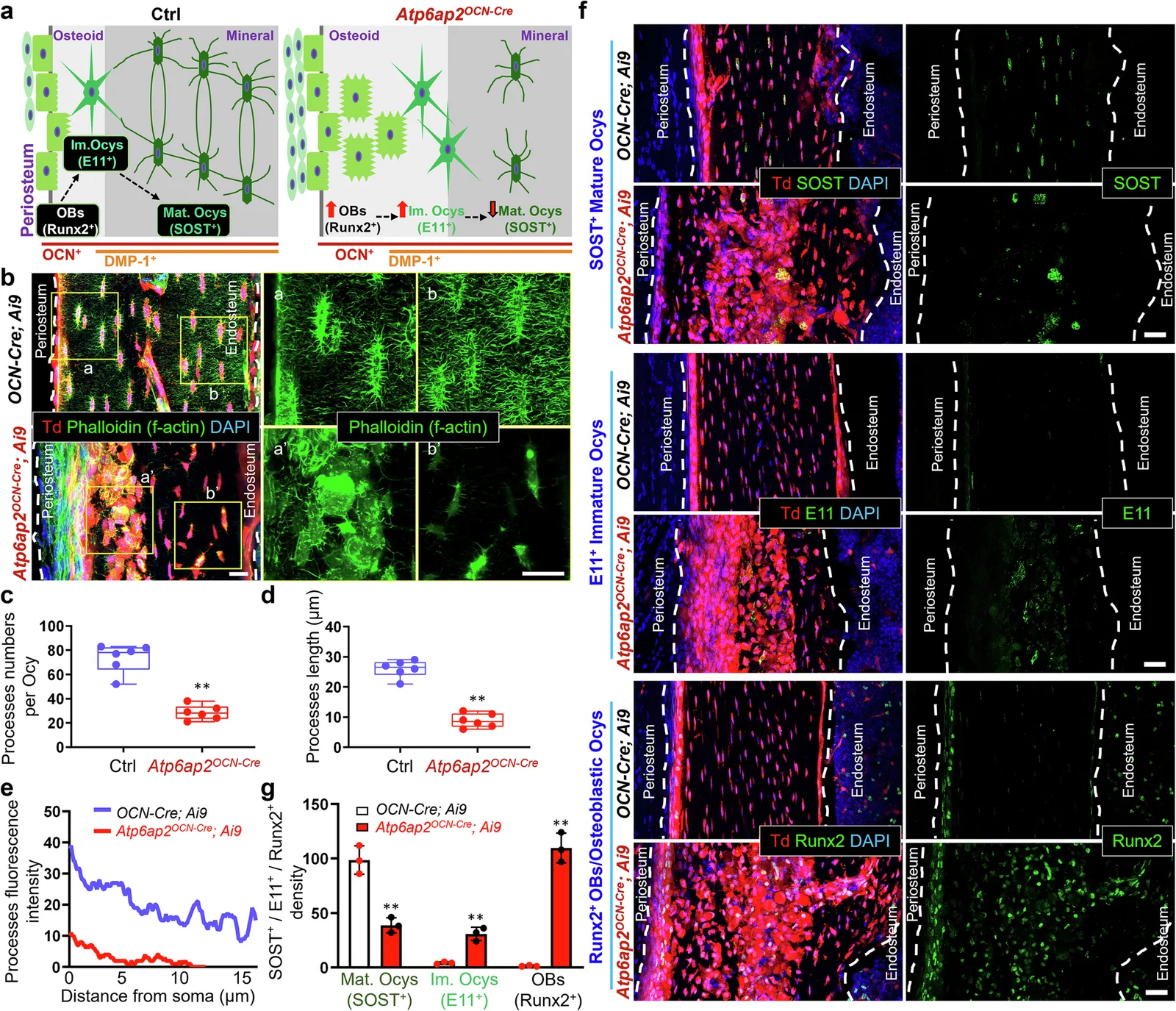
Impaired osteocyte processes and osteocyte maturation in the cortical bone of P30 *Atp6ap2*^*OCN-Cre*^ mice. **a** Schematic diagram depicting the transitional stages that occur as osteoblasts differentiate into mature osteocytes. The volume of the cell body and the number of cell organelles decrease during this process. **b** Immunofluorescence labeling of osteocytes with phalloidin-488 for f-actin (green) in femur cortical bone (diaphysis) of P30 mice with targeted deletion of *Atp6ap2* in cells expressing osteocalcin (TdTomato^+^, *Atp6ap2*^*OCN-Cre*^*; Ai9*) and their littermates (*OCN-Cre; Ai9*). Right panel shows magnification of yellow boxed area of left panels. Bar, 20 μm. **c–e** Quantifications of the processes number per osteocytes (**c**), the processes length (**d**) and the processes fluorescence intensity (**e**). **f** Immunofluorescence of femur cortical bone (diaphysis) in P30 male *OCN-Cre; Ai9* and *Atp6ap2*^*OCN-Cre*^*; Ai9* mice using SOST (mature osteocytes marker), E11 (also named gp38, early immature osteocyte marker) and Runx2 (preosteoblast, osteoblast, osteoblastic osteocyte marker) antibodies. Bar, 50 μm. **g** Quantifications of the density of SOST^+^, E11^+^ and Runx2^+^ cells. The values of mean ± SD (*n* = 3 animals per genotype) were shown. Data in (**c**, **d**) are shown as box plots with individual data points. The center line indicates the median, the box indicates the interquartile range, and whiskers indicate minimum to maximum values (*n* = 6 animals per genotype). Data in (**g**) is shown as mean ± SD (*n* = 3 animals per genotype). *P* values obtained by unpaired two-tailed t-test. **P < 0.01.

Given the marked alterations in osteocyte distribution and morphology in the mutant mice (Fig. 3a–g), we further examined the detailed osteocyte phenotypes. First, mature osteocytes have long cytoplasmic extensions or processes, which occupy canaliculi in the matrix and are used for exchange of nutrients and wastes through gap junctions^14,16^ (Fig. 4a). To examine these processes, we used phalloidin labeling, a marker of the osteocyte processes^7^. As shown in Fig. 4b, abundant well interconnected osteocyte processes were detected in the control (*OCN-Cre; Ai9*, P30) cortical bone sections. These processes appeared to be evenly distributed, without overlapping each other, exhibiting a feature of “tiling” (Fig. 4b–e). However, in the mutant (*Atp6ap2*^*OCN-Cre*^*; Ai9*, P30) mice, little to no F-actin^+^ osteocyte processes were detected (Fig. 4b–e), demonstrating a severe disruption in this event in the mutant mice, and implicating ATP6AP2’s function in promoting osteocyte morphogenesis and/or maturation.

Mature osteocytes [marked by SOST (Sclerostin)] are terminally differentiated cells from the OB-lineage precursors, which include Runx2 (Runt-related transcription factor 2)^+^ OBs (also called osteoblastic osteocytes) and E11/Gp38^+^ immature osteocytes^7,36^ (Fig. 4a). To investigate whether ATP6AP2 promotes osteocyte maturation, we thus examined SOST^+^ mature osteocytes, E11/Gp38^+^ immature osteocytes, and Runx2^+^ OBs/osteoblastic osteocytes in the cortical regions of P30 Ctrl (*OCN-Cre; Ai9*) and mutant (*Atp6ap2*^*OCN-Cre*^*; Ai9*) mice. As shown in Fig. 4f, SOST^+^tdTomato^+^ mature osteocytes were mainly distributed in the central region of the cortical bone in ctrl mice; and these SOST^+^tdTomato^+^ osteocytes were marked reduced in the cortical region of *Atp6ap2*^*OCN-Cre*^*; Ai9* mice (Fig. 4f, g), supporting the view for a decreased osteocyte maturation. In contrast, the E11^+^tdTomato^+^ immature osteocytes were increased and abnormally distributed in the mutant cortical bones (Fig. 4f, g). Further examination of Runx2^+^tdTomato^+^ OBs/osteoblastic osteocytes also revealed a marked increase of these cells in the center area of the mutant cortical bones (Fig. 4f, g), with an altered distribution pattern (Fig. 4f). Collectively, these results suggest a necessity of ATP6AP2 in OB-lineage cells for osteocyte distribution, differentiation/maturation, and morphogenesis.

### Similar, but weaker, cortical bone and osteocyte deficits in *Atp6ap2*^*DMP1-Cre*^ mice

To further investigate the functions of ATP6AP2 in the OB-lineage cells in regulating osteocyte distribution and differentiation, we generated *Atp6ap2*^*DMP1-Cre*^ mice, by crossing *Atp6ap2*^*flox/X*^ mice with DMP1-Cre, which is believed to express Cre largely in mature osteocytes^7,36–39^. The td-Tomato^+^ fluorescence in *DMP1-Cre; Ai9* cortical bones was largely detected in osteocytes, and also muscles (Fig. S3a). Examining osteocyte distribution showed majority of tdTomato^+^ osteocytes to be evenly distributed in the cortical bone in *Atp6ap2*^*DMP1-Cre*^ mice (Fig. S3a, b), unlike that in *Atp6ap2*^*OCN-Cre*^ mice, which showed large number of tdTomato^+^ cells accumulated close to the periosteum side. These results suggest a little to weak deficit in osteocyte distribution when ATP6AP2 was depleted in DMP1-Cre^+^ osteocytes. Further examining osteocyte phenotype showed similar, but also weaker, cortical bone or osteocyte phenotypes, in *Atp6ap2*^*DMP1-Cre*^ mice to those in ATP6AP2^OCN-Cre^ mice. These cortical bone phenotypes in *Atp6ap2*^*DMP1-Cre*^ mice include increases in the cell density and bone thickness (Fig. S3c, d); reduced SOST^+^ mature osteocytes, and increased E11^+^ and Runx2^+^ cells, which were all less server than those in *Atp6ap2*^*DMP1-Cre*^ mice (Fig. S3e, f). Finally, imaging analysis of phalloidin/DAPI marked bone sections showed a decrease in the number of osteocyte processes, without significant change in their length, in *Atp6ap2*^*DMP1-Cre*^*; Ai9* mice, as compared with those of control mice (Fig. S3g–j). These results suggest that ATP6AP2 in DMP1-Cre^+^ mature osteoblasts and osteocytes is required for osteocyte maturation and the maintenance of the osteocyte network, but not osteocyte distribution, implicating a critical role of ATP6AP2 in OCN-Cre^+^ OB/osteocyte precursors for osteocyte distribution, morphogenesis, and maturation.

### Impaired Wnt/β-catenin signaling selectively in the OBs of trabecular bone, but not osteocytes of cortical bone, of *Atp6ap2*^*OCN-Cre*^ mice

ATP6AP2 has been identified as an important modulator of Wnt signaling^26–28^, a critical pathway for bone remodeling^40^. To investigate whether ATP6AP2 regulates cortical bone remodeling through this pathway, we first performed immunostaining using antibody against active β-catenin (Ser33/37/Thr41 non-phospho β-catenin) on femur sections from P30 control (OCN-Cre; Ai9) and ATP6AP2^OCN-Cre^; Ai9 mice. Consistent with previous studies^28^, we observed a marked reduction in active β-catenin signals in the trabecular bone region of *Atp6ap2*^*OCN-Cre*^*; Ai9* mice (Fig. S4a, b), supporting the view for ATP6AP2 to promote trabecular bone formation by maintaining Wnt/β-catenin signaling activity^28^. However, in cortical bone, a distinct pattern was observed. While ATP6AP2 deletion reduced active β-catenin levels in the periosteal region, likely to be osteoblastic osteocytes (Fig. S4a, c), the β-catenin levels were increased in the cortical bone enlarged tdTom-positive cells in the mutant mice (Fig. S4a, c). These findings suggest that ATP6AP2 differentially regulates Wnt/β-catenin signaling in cortical versus trabecular bone, suggesting context-dependent roles in bone remodeling.

To further test above view, we crossed *Axin2*^*LacZ*^ mice, a Wnt/β-catenin signaling LacZ reporter mouse line, with *Atp6ap2*^*OCN-Cre*^ to generate *Axin2*^*LacZ*^*; Atp6ap2*^*OCN-Cre*^ mice. X-gal staining analysis revealed abundant β-galactosidase activity in the trabecular region of the ctrl mice (*Axin2*^*LacZ*^*; OCN-Cre*), which was marked reduced in the mutant mice (Fig. S4d, e). Notably, β-galactosidase activity was low in the cortical diaphysis of both control and mutant mice, with no obvious difference observed between the two groups (Fig. S4d, f). These results provide in vivo evidence that ATP6AP2 is required for Wnt/β-catenin signaling pathway in osteoblasts within the trabecular bone and periosteum of cortical bone. Its role in regulating Wnt/β-catenin signaling in osteocytes of cortical bone remains to be further elucidated.

### Increased osteoclastic bone resorption that contributes to the decreased trabecular bone-mass, but not cortical bone-mass, in *Atp6ap2*^*OCN-Cre*^ mice

Lack of osteoclasts or defective osteoclast activity could result in increased bone mass^5^. We thus asked whether osteoclastic bone resorption is altered and thus contributes to the increased cortical bone-mass in *Atp6ap2*^*OCN-Cre*^ mice. However, TRAP staining showed increased TRAP^+^ osteoclast numbers in both trabecular and endo-cortical bone surfaces in P90 *Atp6ap2*^*OCN-Cre*^ mice (Fig. S5a, b). Measuring serum levels of PYD (a marker for bone resorption) also demonstrated elevated bone resorptive activity in the mutant mice at the age of P30 and P90 (Fig. S5c). These results suggest a negative role of osteoblastic ATP6AP2 in regulating osteoclast activity, implicating a contribution of osteoclast mediated bone remodeling to the phenotypes in the mutant mice.

We further tested the latter view by treating the control and mutant mice with bisphosphonates (alendronate), an inhibitor of osteoclast activity (Fig. S5d). Measuring serum levels of PYD verified alendronate’s suppressive effect on bone resorption in both control and *Atp6ap2*^*OCN-Cre*^ mice (Fig. S5e). Interestingly, alendronate treatment significantly increased both trabecular and cortical bone mass in the ctrl mice; however, in the mutant mice, the treatment only restored the trabecular bone-mass, but had little effect on the cortical bone phenotype (Fig. S5f–i). These results thus suggest that increased osteoclasts contribute to the reduced trabecular bone-mass, but had minimal effect on the cortical bone defects in the mutant mice.

### Reduced MMP14 surface distribution and activity in *Atp6ap2*-KO OBs and immature osteocytes

**Fig. 5:**
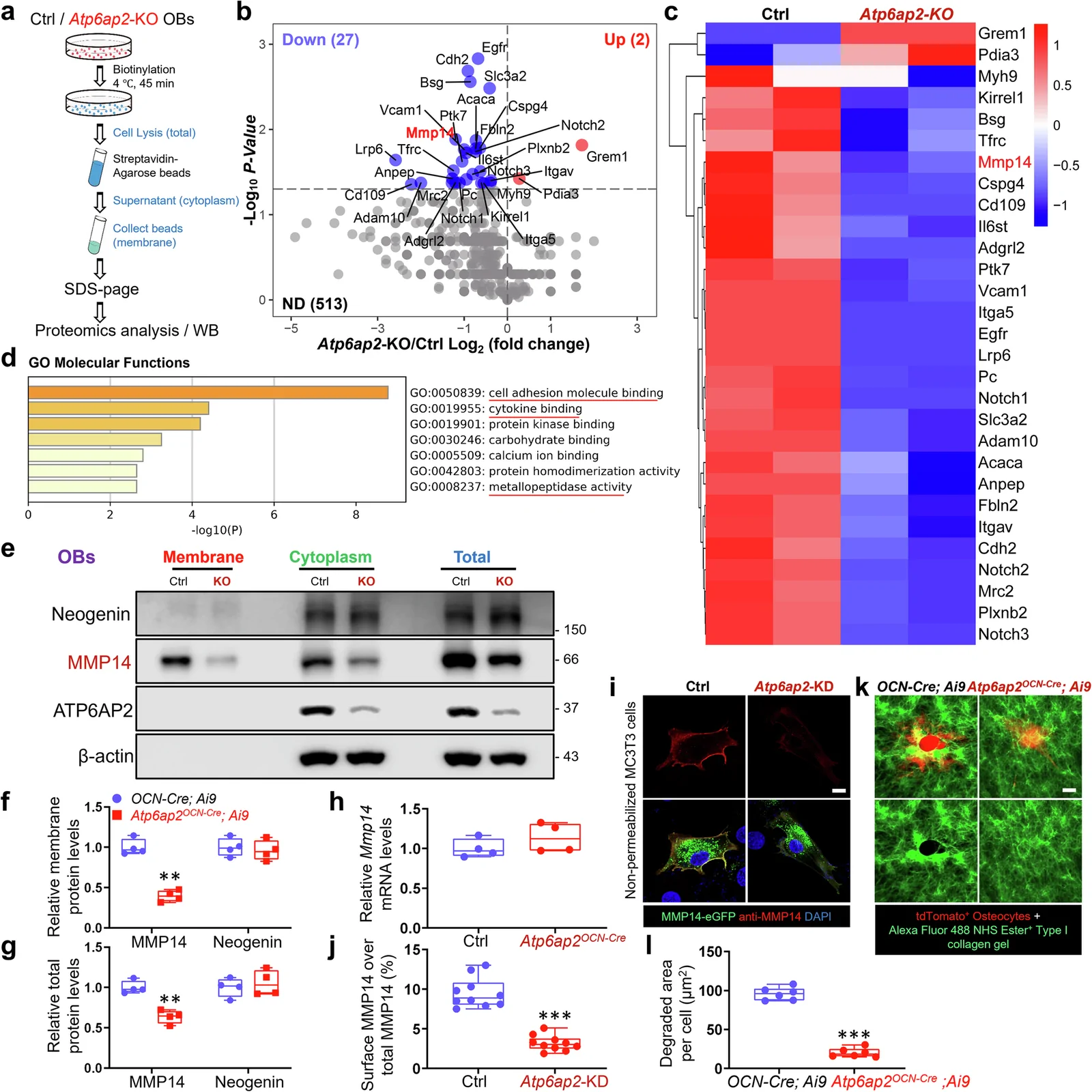
Reduced MMP14 surface distribution and activity in *Atp6ap2*-KO osteoblasts and immature osteocytes. **a** Schematic diagram of quantitative proteomic analysis. Proteomic data were obtained from surface proteins of ctrl and *Atp6ap2*-KO OBs. **b** Volcano plots of differentially expressed proteins in the cell surface of ctrl and *Atp6ap2*-KO OBs. The up-regulated proteins were marked in red, and the down-regulated proteins were indicated in blue (*P* < 0.05). **c** Heat map protein expression z-scores computed for the 29 proteins that are differentially expressed in cell membrane of Ctrl and *Atp6ap2*-KO OBs (*P* < 0.05). **d** The gene ontology (GO) molecular functional analysis shows the top 7 significant enrichment terms represented by molecular function, with longer column representing more significant enrichment. MMP14 was involved in many biological processes, including cell adhesion molecule binding, cytokine binding and metallopeptidase activity. **e–g** Western blot analyses of lysates of total, cytoplasm and cell surface fractions of ctrl and *Atp6ap2*-KO OBs. Quantification of the data was shown in (**f**, **g**). **h** Real-time PCR analysis of *Mmp14* expression in BMSCs of P90 ctrl and *Atp6ap2*^*OCN-Cre*^ mice. **i**, **j** Immunostaining analysis of cell surface MMP14 in non-permeabilized ctrl and *Atp6ap2*-KD MC3T3 cells transfected with the MMP14-eGFP plasmid. Bar, 10 µm. Quantification analysis was shown in (**j**), the ratio of cell surface MMP14 over total MMP14. **k**, **l** Representative images (**k**) and quantitative measurement (**l**) of type I collagen layer degradation assay of osteocytes derived from *OCN-Cre; Ai9* and *Atp6ap2*^*OCN-Cre*^*; Ai9* mice. The type I collagen gel was labeled by Alexa fluor 488 NHS ester dye before seeding the cells. Confocal micrographs show degradation of the fluorescent collagen layer, indicated by a dark hole, in ctrl osteocytes, but not in *Atp6ap2*-KO osteocytes. Bar, 10 μm. Data in (**f**–**h**), (**j**) and (**l**) are shown as box plots with individual data points. The center line indicates the median, the box indicates the interquartile range, and whiskers indicate minimum to maximum values (*n* = 4 in (**f**–**h**), *n* = 10 cells in (**j**), *n* = 6 in (**l**)). *P* values obtained by unpaired two-tailed *t* test. **P < 0.01. ***P < 0.001.

To investigate molecular mechanisms underlying osteoblastic ATP6AP2 regulated cortical bone development, we carried out proteomic analysis by liquid chromatography-tandem mass spectrometry to screen for altered plasma membrane proteins in primary cultured *Atp6ap2*-KO OCN-Cre^+^ OB-lineage cells (Fig. 5a). Both ctrl (OCN-Cre) and *Atp6ap2*-KO OB-lineage cells were incubated with NHS-biotin for 45 min at 4 °C to label cell surface membrane proteins. The biotin labeled surface proteins were pulled down by streptavidin-agarose beads and subjected to proteomic analysis and western blot analysis. Among a total of 542 proteins identified, 27 proteins were down-regulated and 2 proteins were up-regulated in *Atp6ap2*-KO OBs, as compared with those of ctrl (Fig. 5b). Heat map analysis of these 27 downregulated and 2 upregulated proteins was illustrated in Fig. 5c. Gene ontology (GO) molecular functional analysis of these dysregulated proteins exhibited different biological processes, including cell adhesion molecules, cytokine binding proteins, and metallopeptidase activity (Fig. 5d). We further investigated MMP14, also called MT1-MMP, a protein belonging to the matrix metalloproteinase (MMP) family that is critical for the breakdown of extracellular matrix (ECM)^41,42^. MMP14 was indeed markedly reduced in the plasma membrane fractions in *Atp6ap2*-KO OBs, as compared with that of controls, by Western blot analysis of biochemical cell fractions of OBs (Fig. 5e, f). As a control, neither the membrane-associated nor the total levels of Neogenin (a DCC family receptor) were altered (Fig. 5e–g). Notice that the total protein levels of MMP14 was also reduced in *Atp6ap2*-KO cells (Fig. 5e, g), while its mRNA level had little to no change (Fig. 5h), suggesting that ATP6AP2 may play a role in regulating MMP14 protein trafficking and stability. This view was further supported by the observations of lower cell surface levels of exogenous expressed MMP14-eGFP in *Atp6ap2*-KD (knockdown) MC3T3 cells than those of control MC3T3 cells (Fig. 5i, j). As MMP14 surface distribution is crucial for its activation, we further tested this view by examining MMP14 mediated degradation of the extracellular matrix (ECM)(e.g., type I collagen) in control (*OCN-Cre; Ai9*) and mutant (*Atp6ap2*^*OCN-Cre*^*; Ai9*) mice. As expected, the degradation of the fluorescent conjugated type I collagen was much lower in osteocytes derived from the mutant mice than those of control mice (Fig. 5k, l), indicating a reduced MMP14 activity in *Atp6ap2*-KO osteocytes.

We next determined whether in vivo MMP14 expression and distribution in osteocytes of cortical bones were affected by loss of ATP6AP2. Notice that MMP14 was abundantly expressed in osteocytes that are largely distributed in the periosteum region of the control mice (*Ai9; OCN-Cre*) (Fig. S6a–f), implying MMP14 to be selectively expressed or enriched in immature or osteoid osteocytes or osteoblastic osteocytes. Indeed, co-immunofluorescence labeling analysis showed that MMP14^+^ osteocytes were E11^+^, a marker for immature osteocytes (Fig. S6g, h). In contrast from the control mice, MMP14 in *Atp6ap2*^*OCN-Cre*^*; Ai9* mice was marked decreased and abnormally distributed, as those of E11^+^ immature osteocytes in the mutant mice (Fig. S6g, h). MMP14 was reduced in E11^+^ immature osteocytes in the mutant cortical bones (Fig. S6g, h). Higher power imaging analysis showed a vesicle-like distribution of MMP14 in the mutant osteocytes, instead of an even cell surface distribution in the ctrl osteocytes (Fig. S6d–f). These results thus provide in vivo evidence for ATP6AP2 to regulate MMP14 protein trafficking and surface targeting, likely in large in immature osteocytes.

### ATP6AP2 interacting with MMP14 and increasing MMP14 surface targeting

To address how ATP6AP2 regulates MMP14 protein trafficking and surface targeting, we performed co-immunofluorescence labeling analyses of MMP14 with endosome/lysosome markers in control and *Atp6ap2*-KD MC3T3 cells. A significantly reduced MMP14 co-localization with EEA1 (a marker for early endosomes) and Rab7 (a marker for late endosomes), but an increased MMP14 co-localization with LAMP1 (a marker for early lysosomes) were detected in *Atp6ap2*-KD MC3T3 stable cell line (an OB cell line) (Fig. S7a, b), suggesting a role of ATP6AP2 in suppression of MMP14 targeting to the LAMP1^+^ lysosomes, but not endosomes, and implicating ATP6AP2’s function in preventing MMP14 degradation. We then tested the latter view by examining MMP14’s half-life. Indeed, a faster protein turnover of MMP14 was detected in *Atp6ap2*-KD MC3T3 cells, exhibiting a half-life of ~1 hr in *Atp6ap2*-KD, but ~2-hr in control cells, (Fig. S7c, d).

**Fig. 6:**
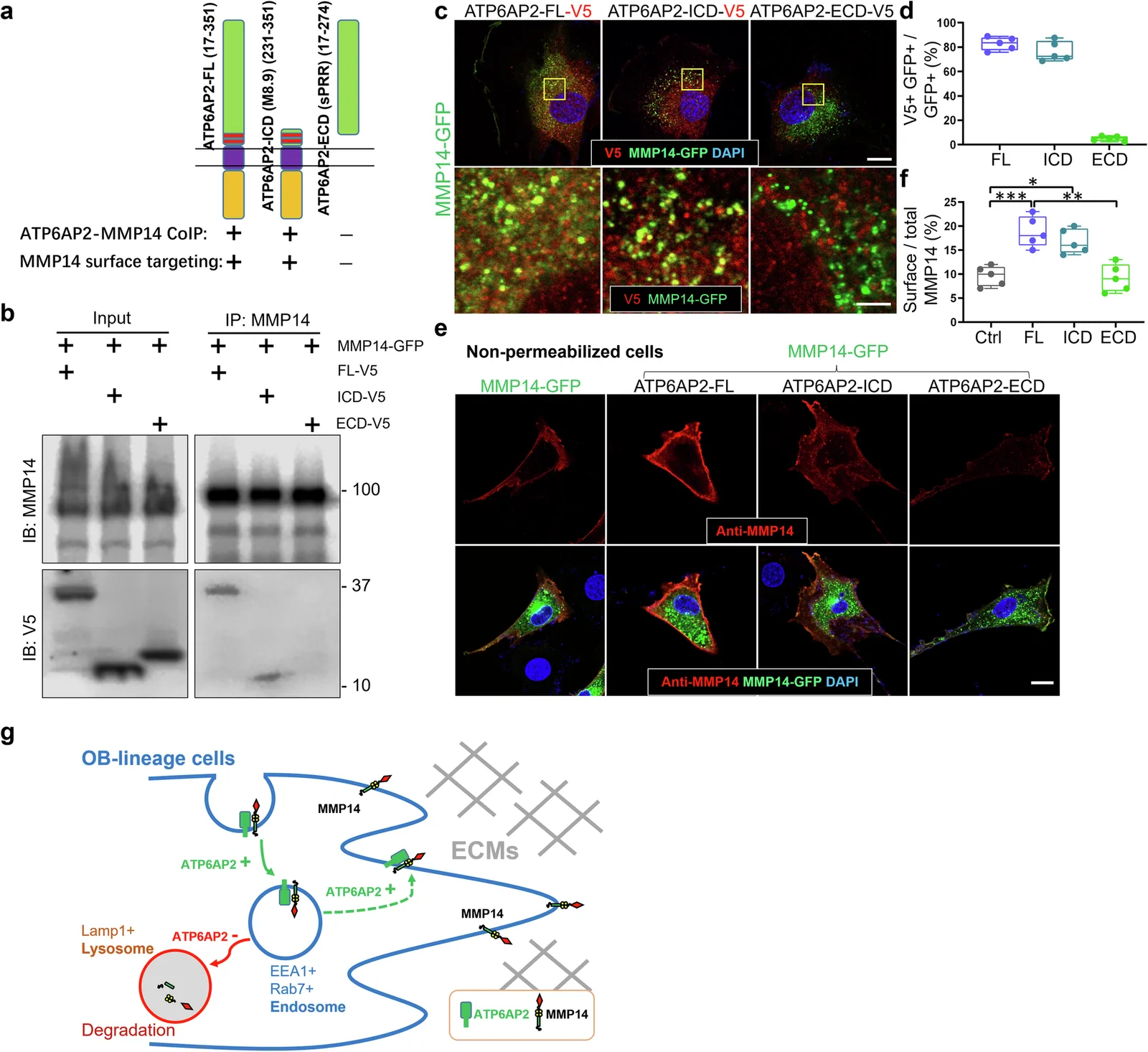
ATP6AP2 promotes MMP14 surface targeting by interacting with MMP14. **a** Illustration of various deletion mutants of ATP6AP2. **b** Coimmunoprecipitation analysis of ATP6AP2 and its mutant with MMP14. HEK293T cells were transfected with the indicated plasmids. 48 h after transfection, ∼500 µg cell lysates was immunoprecipitated by anti-MMP14 antibodies and A/G agarose. The resulting lysates were subjected to Western blot analysis using indicated antibodies. Approximately 50 µg cell lysates were used as an input. Data presented are representative of three independent experiments. **c**, **d** Co-immunostaining analysis of MMP14-eGFP with various deletion mutants of ATP6AP2 in MC3T3 cells. Representative images are shown in (**c**). Bar, 10 μm (single cell) and 2 μm (zoom). Quantification analysis were shown in (**d**). The co-localization rates of various mutants of ATP6AP2 with MMP14 were determined by the measurement of overlapped signaling (yellow fluorescence) over total GFP^+^ signal. **e**, **f** Immunostaining analysis of cell surface MMP14 in non-permeabilized MC3T3 cells transfected with MMP14-eGFP and various deletion mutants of ATP6AP2. Bar, 10 µm. Quantification analysis of the ratio of cell surface MMP14 over total MMP14 was shown in (**f**). **g** Illustration of a working model in which *Atp6ap2* deficiency in OB-lineage cells damage MMP14 trafficking in Rab7^+^ vesicle, consequently reducing cell surface levels of MMP14, which leads to disruption of collagen cleavage in osteocytes and ultimately to the loss of formation of osteocyte processes. Data in (**d**, **f**) are shown as box plots with individual data points. The center line indicates the median, the box indicates the interquartile range, and whiskers indicate minimum to maximum values (five-different assays). *P* values obtained by one-way ANOVA followed by Tukey post hoc test. *P < 0.05. **P < 0.01. ***P < 0.001.

To further understand how ATP6AP2 regulates MMP14 surface distribution, we wondered whether both proteins form a complex. To this end, HEK293 cells were co-transfected plasmids encoding MMP14-GFP with plasmids encoding V5 tagged ATP6AP2 -full length (FL), and its deletion mutants- the intracellular domain (ICD) (M8.9) and the soluble form or N-terminal domain (ECD) (Fig. 6a). Resulting cell lysates were subjected to immunoprecipitation with anti-MMP14 antibodies. Interestingly, the ATP6AP2-FL and ICD/M8.9, but not the ECD, were detected in the MMP14 immuno-precipitates (Fig. 6b), suggesting that ATP6AP2 interacts with MMP14 via its ICD/M8.9. This view was further supported by the observations of co-localization of MMP14-eGFP with ATP6AP2-FL or ICD/M8.9, but not ECD, by co-immunostaining analysis (Fig. 6c, d). We next examined whether expressing these ATP6AP2-FL and mutant proteins could regulate the cell surface distribution of MMP14-eGFP in MC3T3 cells. As shown in Fig. 6e, f, expressing ATP6AP2-FL and ICD/M8.9, but not the ECD, increased the surface levels of MMP14-eGFP. These results thus suggest a critical role of the ICD domain of ATP6AP2 in forming a complex with MMP14 and in promoting MMP14’s cell surface distribution, supporting the view for ATP6AP2 to promote MMP14 surface targeting by interacting with MMP14 (Fig. 6g).

### MMP14 attenuation of the cortical osteocyte deficits in *Atp6ap2*^*OCN-Cre*^ mice

To determine whether the reduced MMP14 is responsible for the cortical bone deficit in *Atp6ap2*^*OCN-Cre*^ mice, it is necessary to address if expression of MMP14 into the mutant mice is capable to rescue the deficits. We thus generated lentivirus expressing MMP14-eGFP fusion protein in a Cre-dependent manner (Fig. S8a, b). Primary cultured OBs from *Atp6ap2*^*OCN-Cre*^ mice infected with the lentivirus-MMP14-eGFP showed MMP14-eGFP fusion protein expression (Fig. S8c). As MMP14 surface targeting is crucial for its activation, we further tested this view by examining MMP14 mediated degradation of the extracellular matrix (ECM) (e.g., type I collagen). Control and *Atp6ap2*-KO OBs infected with or without lentivirus-MMP14-eGFP were cultured with fluorescence conjugated type I collagen gel. Whereas *Atp6ap2*-KO OBs showed a decrease in the collagen degradation, as compared with that of control OBs, infection of the lentivirus-MMP14-eGFP in the mutant OBs could diminish the deficit (Fig. S8d, e), supporting the view for ATP6AP2-MMP14 pathway in regulating collagen matrix degradation in culture.

**Fig. 7:**
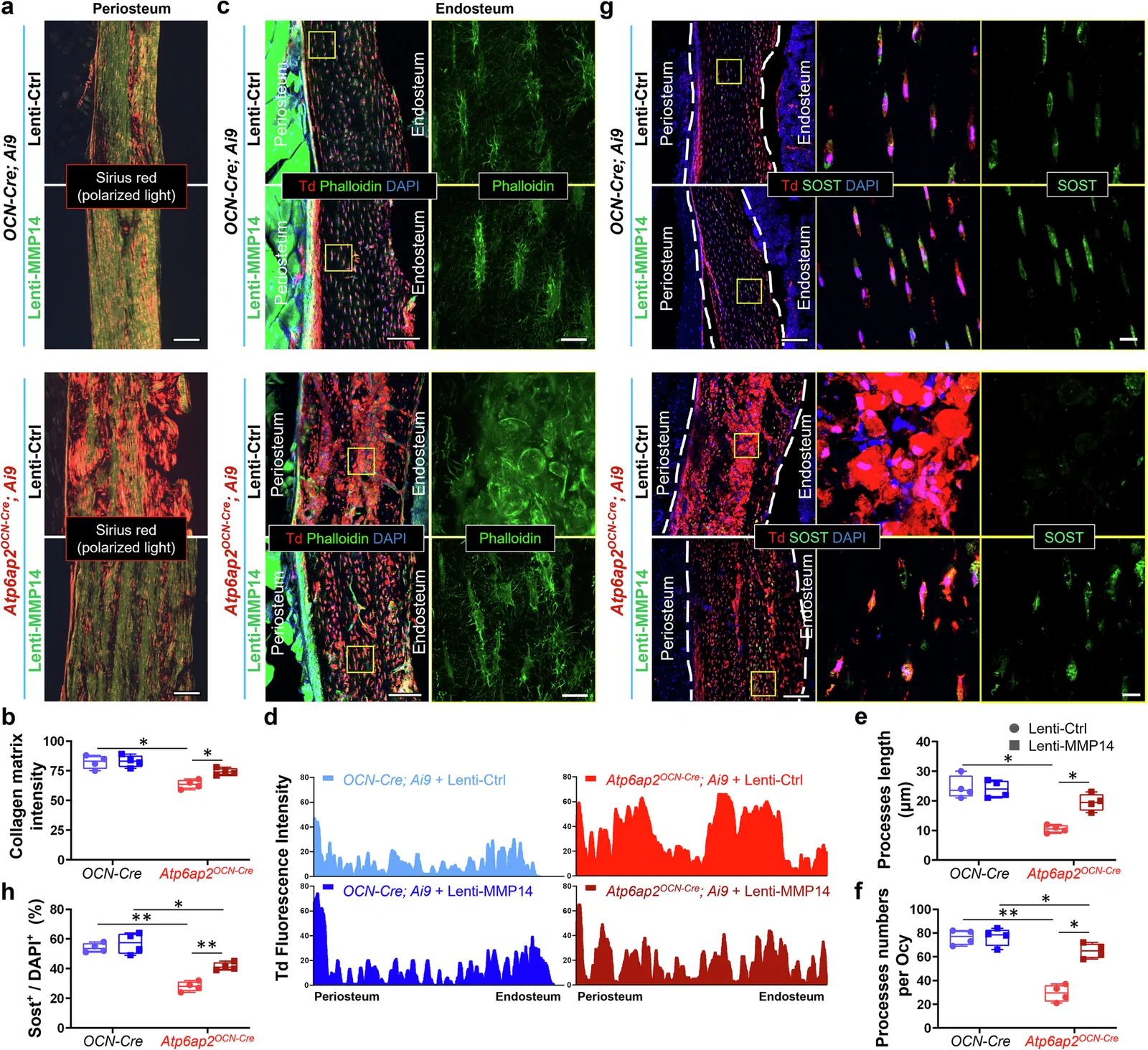
MMP14 attenuation of the cortical osteocyte deficits in *Atp6ap2*^*OCN-Cre*^ mice. **a**, **b** Representative images of Sirius red stained cortical bone (diaphysis) of indicted mice at the age of P45. Bar, 100 μm. Quantification of collagen matrix intensity was shown in (**b**). **c** Immunofluorescence labeling of osteocyte processes with phalloidin-488 for f-actin (green) in femur cortical bone (diaphysis) of indicted mice with ctrl or MMP14-eGFP expression. Bar, 100 μm in the main images and 20 μm in the boxed enlarged views. **d**–**f** Quantification of the TdTomato red fluorescence intensity (**d**), processes length (**e**) and processes number per osteocyte (**f**). **g**, **h** Immunofluorescence labeling of osteocytes with SOST in femur cortical bone (diaphysis) of P45 *OCN-Cre; Ai9* and *Atp6ap2*^*OCN-Cre*^*; Ai9* mice with ctrl or MMP14-eGFP expression. Bar, 100 μm in the main images and 20 μm in the boxed enlarged views. Quantification were shown in (**h**). Data in (**b**, **e**, **f**, and **h**) are shown as box plots with individual data points. The center line indicates the median, the box indicates the interquartile range, and whiskers indicate minimum to maximum values (*n* = 4 animals). *P* values obtained by two-way ANOVA followed by Bonferroni’s post hoc test. *P < 0.05. **P < 0.01.

We then asked whether injecting the lentivirus-MMP14-eGFP into *Atp6ap2*^*OCN-Cre*^ cortical bones could diminish their bone deficits. The control and MMP14-eGFP lentiviruses were injected into the femur cortical bones of control and *Atp6ap2*^*OCN-Cre;*^ *Ai9* mice at age of P15 (Fig. S9a). One month later, the cortical bones were isolated and subjected to the co-immunostaining analysis. As shown in Fig. S9b, in the MMP14-EGFP virus injection side, GFP positive signals were detected in the tdTomato^+^ osteocytes, demonstrating the MMP14-eGFP expression. While no significant changes were detected on control osteocytes expressing MMP14-eGFP fusion protein (Fig. 7a–h), expressing MMP14-eGFP into the *Atp6ap2*^*OCN-Cre*^ osteocytes did attenuate the cortical bone deficits (Fig. 7a–h). The cortical osteocyte phenotypes, including increased disorganized collagen fibers area, reduced F-actin marked osteocyte networks, altered clustered osteocyte distribution, enlarged osteocyte cell body, impaired SOST^+^ mature osteocytes, and increased cleaved caspase-3^+^ apoptotic cells, were all diminished by injecting MMP14-eGFP virus (Figs. 7a–h and  S10a, b). These results suggest that MMP14 is a key downstream protein of ATP6AP2, regulating the cortical osteocyte development.

## Discussion

**Fig. 8:**
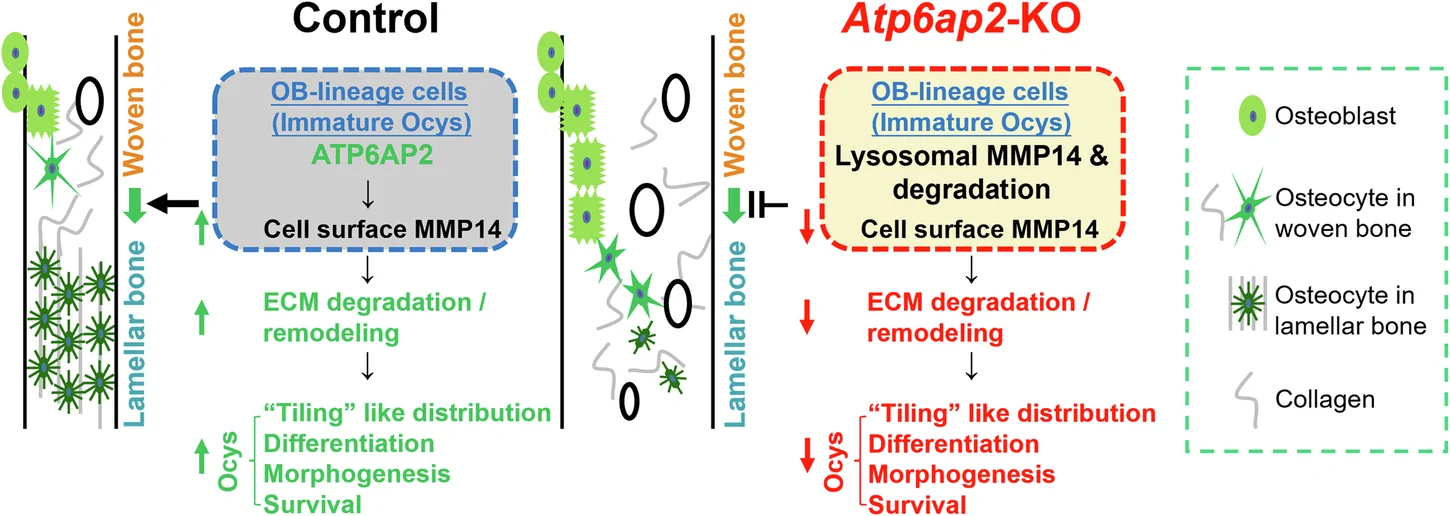
Illustration of a working model. Illustration of a working model in which ATP6AP2 deficiency in OB-lineage cells interrupt MMP14 trafficking, reduce cell surface MMP14 distribution, and thus inhibit MMP14 mediated ECM degradation and remodeling, alter osteocytes distribution, reduce osteocytes differentiation, morphogenesis and survival, and impair woven bone-to-lamellar bone transition.

The present study provides evidence for ATP6AP2 in OCN-Cre^+^ OB-lineage cells to be critical for woven bone-to-lamellar bone transition, and osteocyte development, distribution, and network building. This study also uncovers ATP6AP2’s functions in promoting lamellae bone formation likely by increasing MMP14 surface targeting and activation in immature osteocytes. Further cellular studies lead to a working model that is depicted in Fig. 8. In this model, ATP6AP2 increases MMP14 surface targeting largely from endosomes by interacting with MMP14, and thus enhances MMP14 mediated ECM degradation and remodeling, woven bone-to-lamellar bone transition, osteocyte precursor cells’ distribution, osteocytes differentiation, morphogenesis and survive. This study not only identifies ATP6AP2 as a key regulator of MMP14, but also reveal a pathway from ATP6AP2 to MMP14 in promoting osteocyte development.

The expression levels of ATP6AP2 in osteocytes appeared to be similar to that in osteoblasts and BMSCs (Fig. S1). The phenotype of osteocyte *Atp6ap2* knockout mice (*Atp6ap2*^*DMP-Cre*^) appeared to be weaker than that of the BMSCs/osteoblasts *Atp6ap2* knockout mice (*Atp6ap2*^*OCN-Cre*^) led us to speculate that ATP6AP2 in OCN-Cre^+^ osteoblastic cells (e.g., immature osteocytes) may play a more critical role than that in DMP1-Cre^+^ mature osteocytes in regulating cortical bone matrix remodeling and osteocyte development.

Consistent with this interpretation, dynamic histomorphometry analysis using calcein double labeling demonstrated that mineral apposition rate and bone formation rate were all increased in *Atp6ap2*^*OCN-Cre*^ cortical bone (Fig. 3). These results indicate that osteoblast activity and bone formation are elevated in the absence of ATP6AP2. However, this increased bone formation is not accompanied by proper lamellar bone maturation, but instead correlates with retention of woven bone, increased cortical porosity, and defective osteocyte organization, suggesting a decoupling between bone formation and bone maturation processes.

ATP6AP2 is known to play important roles in multiple signaling pathways and in multiple organs^19^. In addition to function as a PRR, inducing the conversion of angiotensinogen to angiotensin I and regulating blood pressure^22^, it is a critical regulator of Wnt/β-catenin signaling and V-ATPase by its interaction with Wnt receptors (e.g., LRP6 and frizzled) and V-ATPase membrane proteins^26,27^. By its interaction with LRP6 and frizzled, ATP6AP2 promotes Wnt/β-catenin signaling^26,27^. In line with this view is our proteomic analysis, which identified reduced LRP6 surface targeting in *Atp6ap2*-KO OB-lineage cells (Fig. 5b, c). While our data support a major role for ATP6AP2–MMP14–mediated matrix remodeling, the known function of ATP6AP2 in regulating Wnt/β-catenin signaling further suggests that multiple pathways may converge to control cortical bone development and osteocyte maturation.

How does ATP6AP2 promotes MMP14 surface targeting? Encouraged by the following observations, we propose that ATP6AP2 promotes MMP14 surface targeting by interacting with MMP14 largely in endosomes. First, ATP6AP2 forms a complex with MMP14 via its C-terminal ICD (Fig. 6). This ICD domain, also called M8.9, binds to the V-ATPase complex proteins and promotes V-ATPase complex assemble and activation^43^. Second, in *Atp6ap2*-KD MC3T3 cells, expression of this ICD domain is sufficient to increase MMP14 surface targeting (Fig. 6). Third, in addition to the reduced MMP14 surface targeting in *Atp6ap2*-KD MC3T3 cells, MMP14 in EEA1^+^ or Rab7^+^ endosomes were decreased, but MMP14 in Lamp1^+^ lysosomes were increased, in the mutant cells (Fig. S7). Finally, similar V-ATPase’s complex and cell type specific effects on MMPs’ activities are also observed in different tumor cells^44–46^. Given our results that the surface and total levels of MMP14 were decreased in *Atp6ap2*-KO cells (Fig. 5), we speculate that ATP6AP2 may promote V-ATPase driven vesicle pH and MMP14 surface targeting, but not V-ATPase dependent MMP14 degradation. In line with the view for vesicle pH to be critical for MMP14 surface targeting is the report that CLIC4, a chloride intracellular channel 4 that regulates vesicular HCl and pH, colocalizes with MMP14 in the late endosomes, and promotes MMP14 surface targeting and activation in retinal pigment epithelium (RPE)^47^. In addition, ATP6AP2 deficiency was also reported to impair V-ATPase assembly and subsequent defects in glycosylation and autophagy^48^. Further investigation is needed to address whether ATP6AP2 affects the glycosylation of MMP14.

It is also noteworthy that mutations in ATP6AP2, other subunits of V-ATPase, and MMP14 have been identified in various diseases. There are at least 13 subunits identified to form a functional V-ATPase complex, which consists of the peripheral V_1_ component and membrane-bound V_0_ component^49^. The subunits of V-ATPase are ubiquitously expressed, some subunits also have tissue or cell-specific distributions, so the phenotypes of V-ATPase-related human diseases vary from the nervous system, skin, muscle (X-linked myopathy with excessive autophagy, XMEA), kidney (Distal renal tubular acidosis, dRTA) to skeleton (osteopetrosis and osteoporosis) and other tissues^50^. Whereas it is known that V-ATPase is located on the ruffled border plasma membrane of osteoclasts, regulating extracellular acidification for bone demineralization during bone resorption^50^, its function in OBs or osteocytes remains largely unclear. It is of interest to note a recent report^51^ suggests that osteocytes need to acidify their microenvironment in response to PTHrP in vitro and in vivo, implicating V-ATPase in osteocytes’ function.

Multicentric osteolytic syndromes (MOSs), including multicentric osteolysis, nodulosis, and arthropathy (MONA), Winchester syndrome (WS), and Frank-Ter Haar syndrome (FTHS), are rare multi-system disorders featured in large by skeleton anomalies, which include short stature, craniofacial malformations, reduced bone density, open sutures, bowing of long bones, and joint destruction^41,52–56^. Genetic studies in these patients have identified mutations in multiple genes, including *SH3PXD2B* (SH3 and PX Domains 2B), *MMP14* (matrix metalloproteinase 14) and *MMP2* (which is activated by MMP14)^41,52,53,57–61^. As MMP14 to MMP2 pathway is critical for the degradation of collagen, a key component of extracellular matrix (ECM)^41,42^, these clinical and genetic investigations in both human patients and animal models have pointed out that MOSs represent a phenotypic spectrum defined by defective ECM remodeling. In the mice model, a deficiency of MMP14 leads to disruption of collagen cleavage in osteocytes and ultimately to the loss of formation of osteocyte processes, and MMP14-deficient mice develop dwarfism, arthritis, and connective tissue disease due to inadequate collagen turnover^17,62^.

As a subunits of V-ATPase, ATP6AP2 has been reported to contribute to various pathological events/diseases, such as fibrosis, hypertension, acute kidney injury, cardiovascular disease, cancer, obesity, and various other diseases^19^. Our study demonstrates a critical role of ATP6AP2 in regulating cortical bone matrix remodeling and osteocyte development. We speculate that ATP6AP2, as a key regulator of MMP14, promotes the replacement of woven bone to lamellar bone likely by promoting bone ECM remodeling and osteocyte development. Dysregulation of ATP6AP2 to MMP14 pathway may underlie the pathogenesis of many ATP6AP2/MMP14 involved disorders. We hope to further test this view in future.

## Materials and methods

### Ethics statement

All experimental procedures were approved by the Institutional Animal Care and Use Committee at Case Western Reserve University (IACUC, 2017-0121 and 2017-0115), according to US National Institutes of Health guidelines. We have complied with all relevant ethical regulations for animal use.

### Animals and reagents

The female *Atp6ap2*^*flox/X*^ mice^20^ (kindly provided by Dr. Frederique Yiannikouris, University of Kentucky; and Dr. Genevieve Nguyen, INSERM, France) were crossed with male osteocalcin (OCN)-Cre^31^ (kindly provided by Dr. Tom Clemens, Johns Hopkins Medical School) or DMP1-Cre (Jackson lab, Stock No 023047) transgenic mice to generate OB- or Osteocyte- selective conditional knockout (CKO) mutant mice, *Atp6ap2*^*OCN-Cre*^ or *Atp6ap2*^*DMP1-Cre*^, respectively. Since *Atp6ap2* is X-linked, only male mice were used in this study to ensure a sufficient number of littermates. The mutant mice were in C57BL/6 J mouse background (for more than 6 generations). Unless otherwise indicated, male mice ranging from postnatal day 2 (P2) to postnatal day 90 (P90) were used in this study. The Ai9 and *Axin2*^*LacZ*^ mice were purchased from the Jackson Laboratory (Stock No 007909 and 009120), which are in C57BL/6 J background. All mice were maintained under specific pathogen-free (SPF) conditions in accordance with IACUC guidelines. Animals were housed in individually ventilated cages with a 12 h light/12 h dark cycle at a controlled temperature (22 ± 2 °C) and humidity (40–60%). Mice were group-housed (2–5 animals per cage) with environmental enrichment, including nesting material and shelter. Standard autoclaved chow diet and autoclaved water were provided ad libitum throughout the experimental period. Cage bedding was changed regularly under sterile conditions. All efforts were made to minimize animal suffering and to reduce the number of animals used in accordance with the principles of the 3Rs (Replacement, Reduction, and Refinement). Euthanasia was performed in accordance with IACUC guidelines. Mice were euthanized using one of the following approved methods depending on the experimental requirements. For general tissue collection, animals were anesthetized with isoflurane (1–4%, inhalation) followed by anesthetic overdose, and death was confirmed by secondary physical methods (pneumothorax or decapitation). For carbon dioxide euthanasia, animals were exposed to CO₂ in a controlled chamber followed by confirmation of death using secondary methods (pneumothorax or decapitation). For perfusion-based experiments, mice were deeply anesthetized with isoflurane (1–4%, inhalation) prior to transcardial perfusion, and euthanasia was completed under anesthesia with confirmation of death using secondary methods (pneumothorax or decapitation). All procedures were performed in designated institutional facilities.

Mouse monoclonal antibodies, including β-actin (A1978, Sigma, 1:5000 dilution), Rab7 (ab50533, Abcam, 1:1000 dilution) and V5 (V8012, Sigma-Aldrich, 1:5000 dilution for WB, 1:500 dilution for IF), were used. Rabbit monoclonal antibodies Ki67 (ab16667, Abcam, 1:500 dilution), Active beta-Catenin (Ser33/37/Thr41 non-phospho, Cell signaling, 8814S, 1:200 dilution), MMP14 (ab51074, Abcam, 1:2000 dilution for WB, 1:400 dilution for IF) and Runx2 (ab192256, Abcam, 1:400 dilution) were used. Rabbit polyclonal antibody Cleaved Caspase-3 (9661, Cell signaling technology, 1:300 dilution), ATP6AP2 (HPA003156, Sigma Aldrich, 1:2000 dilution), neogenin (1:1000 for blotting), GFP (Clontech, 8367-1, 1:200 dilution) and EEA1 (ab2900, Abcam, 1:1000 dilution) were used. Goat SOST (AF1589, R&D, 1:200 dilution) antibody, Rat Lamp1 (1D4B-c, DSHB, 1:1000 dilution) antibody and Syrian hamster monoclonal E11(gp38 or gp36, ab11936, Abcam, 1:200 dilution) antibody were used. Alexa Fluor 488 phalloidin (f-actin, A12379, 5 assays/mL) was purchased from Thermo Fisher. Secondary antibodies were purchased from Jackson ImmunoResearch Laboratories, Inc. Cycloheximide solution (CHX, C4859) was obtained from Sigma-Aldrich. Other chemicals and reagents used in this study were of analytical grade.

### Plasmids and lentiviruses

ATP6AP2-V5-FL and ATP6AP2-V5-ICD plasmids were purchased from DNASU (ATP6AP2 in pLX304, HsCD00446844 and HsCD00437681). ATP6AP2-V5-ECD was made by cloning the ECD domain (17–274) of ATP6AP2 into the pLX304 plasmid (KpnI and XbaI). MMP14-eGFP plasmid was purchased from Addgene (item ID 89819). Renin Receptor shRNA Lentiviral Particles (shR-PRR) was obtained from Santa Cruz (sc-62935-V). The authenticity of all constructs was verified by DNA sequencing.

The 3rd generation cre-dependent lentiviral system was used to generate the pLV-FLEX-MMP14-eGFP lentiviral particle. MMP14-eGFP was cloned into pLV-FLEX lentiviral plasmid. The empty ctrl or MMP14-eGFP lentiviral plasmid (7.5 μg) was co-transfected into 293 cells with packaging plasmid pRSV-Rev (5 μg, Addgene #12253), pMDLg/pRRE (2.5 μg, Addgene #12251), and envelope plasmid pMD2.G (0.8 μg, Addgene #12259) to package the lentivirus. The lentivirus was concentrated before use.

### In vitro OB-lineage cell cultures

Whole bone marrow cells were flushed from long bones of male Ctrl and *Atp6ap2*-deficient mice and plated on 100 mm culture plates in DMEM containing 1% penicillin/streptomycin (P/S), 10% fetal bovine serum (FBS) for 2 days. For OB-lineage culture, plates with adherent cells were replaced with fresh culture medium every 3 days. After 7 days, passaging cells (BMSCs) by trypsin digestion, 1 × 10^4^/cm^2^ were plated for experiments.

Primary OB cultures were prepared from long bones of male mice. Briefly, small bone pieces were incubated in collagenase solution to remove all remaining soft tissue and adhering cells, then transfer to 60 mm culture dishes containing DMEM medium supplemented with 10% FBS, 1% penicillin/streptomycin, 10 mM β-glycerophosphate and 50 μM L-ascorbic acid-2-phosphate. Replace culture medium three times per week. Bone cells will start to migrate from the bone chips after 3-5 days. After two weeks, the monolayer is trypsinized by incubating the cells with trypsin solution. OBs were plated on 100 mm tissue culture plates in α-MEM containing 10% fetal bovine serum (FBS), 1% penicillin/streptomycin (P/S).

Primary osteocyte cultures were isolated from mouse long bones as described previously^63^. In brief, long bones (femur and tibia) were aseptically dissected from skeletally mature P90 mice. Collagenase solution was prepared as 300 active U/mL collagenase type-IA (Sigma-Aldrich, St. Louis, MO, USA) dissolved in DMEM. EDTA tetrasodium salt dehydrate (EDTA) solution (5 mM, pH = 7.4; Sigma-Aldrich) was prepared in magnesium and calcium-free Dulbecco’s phosphate-buffered solution (DPBS; Mediatech) with 1% BSA (Sigma-Aldrich). All steps of the digestion took place in 5 mL solution in a six-well plate, on a rotating shaker set to 200 RPM, in a 37 °C and 5% CO_2_ humidified incubator. Following each sequential digestion, the digest solution with suspended cells was removed from the bone pieces and kept. The combined cell suspension solution was spun down at 900 rpm for 5 min, the supernatant was removed from the cell pellet, and cells were resuspended in culture medium and counted.

### Cell line and transfection

MC3T3-E1 and HEK293 cells were derived from ATCC. They were tested negative for mycoplasma. MC3T3-E1 or HEK293 cells were maintained in DMEM supplemented with 10% fetal calf serum and 1% penicillin/streptomycin. For transient transfection, MC3T3-E1 cells were plated at a density of 10^6^ cells per 10-cm culture dish and allowed to grow for 12 hours before transfection by using Lipofectamine™ 3000 Transfection Kit (L3000, Invitrogen). 48 hours after transfection, cells were subjected to immunostaining analysis. HEK293 cells were transfected by Polyethylenimine (PEI), as described previously^30,64^. In brief, 12 μg DNA mixture was prepared in serum-free DMEM, 6 μl PEI (based on a 3:1 ratio of PEI: total DNA) was added to the diluted DNA and mixed immediately by pipetting. After incubation for 20 min at room temperature, the DNA/PEI mixture was added to cells, and 48 hours later, transfected cells were subjected to western blot or CO-IP assay.

The *Atp6ap2*-KD cell line was obtained by infection of MC3T3-E1 cells with lentiviral particles encoding scramble control or shRNA-ATP6AP2, respectively. In brief, cells were infected with the lentiviral particles for 1 day in polybrene (2 μg/ml) medium. At day 3, the culture medium was removed and replaced with complete medium (without polybrene). After 5–6 days, stable clones expressing the shRNA were selected via puromycin dihydrochloride (5 μg/ml) that induces death of the un-transduced cells.

### Cell lysis, western blot, and co-immunoprecipitation

Cells were lysed in lysis buffer containing 50 mM Tris-HCl (pH7.5), 150 mM NaCl, 1%(v/v) Triton X-100, 0.1% SDS, 0.5% deoxycholate and 1 mM EDTA, supplemented with protease inhibitors (1 μg/mL leupeptin and pepstatin, 2 μg/mL aprotinin and 1 mM PMSF) and phosphatase inhibitors (10 mM NaF and 1 mM Na3VO4). Whole cell extracts were fractionated by SDS-PAGE and transferred to a nitrocellulose membrane (Bio-Rad). After incubation with 5% milk in TBST (10 mM Tris, 150 mM NaCl, 0.5% Tween 20, pH 8.0) for 1-h, the membrane was incubated with indicated antibodies overnight at 4 °C. Membranes were washed with TBST for three times and incubated with a 1:5000 dilution of horseradish peroxidase-conjugated anti-mouse or anti-rabbit antibodies for 1-h. Blots were washed with TBST three times and developed with the ECL system (Bio-Rad). Immunoprecipitation was performed with an anti-MMP14 antibody. Western blotting was performed using the ECL procedure according to the manufacturer’s instructions (Bio-Rad), with a mouse monoclonal V5 antibody to detect the co-immunoprecipitation ATP6AP2-V5 or anti-MMP14 rabbit polyclonal antibody for MMP14 proteins.

### Immunofluorescence labeling and imaging analysis

For bone section staining, mouse femur, vertebrate bones or skull bones were fixed with 4% PFA in PBS at 4 °C overnight, then decalcified in 14% EDTA for 10 days. The samples were place in OCT and cut into 50 μm sections on a cryostat (HM550, Thermo Scientific). For cell immunofluorescence labeling, cells on coverslips were fixed with 4% paraformaldehyde at room temperature for 20 min. Bone sections or cells were permeabilized with 0.2% Triton X-100 for 8 min, and then subjected to co-immunostaining analysis using indicated antibodies. For cell surface protein staining, cells were directly subjected to co-immunostaining analysis without Triton X-100 (non-permeabilized). Stained sections or cells were washed 3 times with PBS and mounted with VECTASHIELD (H-1500; Vector Laboratories) and imaged by Confocal Microscope at room temperature. Fluorescent quantification was performed using ZEN software according to the manufacturer’s instructions (Carl Zeiss).

### Micro-computed tomography (μCT)

The μCT analyses were carried out as described previously^29,65^. Briefly, mouse femurs were excised, fixed in 10% formaldehyde for 24 h, and then were scanned using the Siemens Inveon Micro PET/CT scanner (10-μm resolution) or Mediso nanoScan SPECT/CT/PET system. The linear attenuation is converted to mineral density based on a hydroxyapatite calibration standard. Scans were automatically reconstructed into 2D slices, and the 3-D reconstruction was performed using all the outlined slices (Amira 3D). For cortical bone volume analysis, the 3-D reconstruction was performed on 25 slices (each slice was 12 µm in thickness) at the midshaft of the femur. The region of interest (ROI) was drawn on every slice and fitted to the outside of the cortical bone to include all the bone and marrow. The threshold for cortical bone was set at 621 mgHA/cm^3^. Data were obtained on bone volume (BV), total volume (TV), BV/TV, bone density and cortical thickness. For cortical porosity analysis, slices were analyzed from the midshaft femur. Bone volume measurements included void spaces (pores) between the periosteal and endosteal surfaces, with cortical porosity defined as the percentage of void space within the cortical bone region.

### Bone histomorphometric analysis

Bone histomorphometric analyses were carried out as previously described^35,66^. Decalcified paraffin sections were used for H&E, Safranin-O, TRAP, Picro-Sirius red and X-gal staining. In brief, mouse tibia and femurs were fixed overnight in 10% formalin, decalcified in 14% EDTA, embedded in paraffin, sectioned, and subjected for hematoxylin and eosin (H&E), TRAP, Picro-Sirius red staining, and Safranin-O analyses which were counterstained by fast green. Brightfield and polarized microscopic images were obtained (BZ-X710 microscope with a pair of circular polarizing filters, Keyence) and analyzed using ImageJ. For the picro-sirius red staining, polarized images were used to measure woven bone area and brightfield images were used to measure cortical area. In brightfield microscopy collagen is red on a pale yellow background. Under polarized light, the larger collagen fibers are deep red, bright yellow or orange, and the thinner ones are green. For X-gal staining, femur sections were washed in PBS and incubated with X-gal staining solution [1 mg/mL X-gal, 5 mmol/L K_3_Fe(CN)_6_, 5 mmol/L K_4_Fe(CN)_6_, 2 mmol/L MgCl_2_ in PBS] at 37 °C overnight. Following several washes in PBS, then counterstained with 0.2% eosin. Slides were mounted with the aqueous mounting medium before taken the images.

### Dynamic bone histomorphometry to measure the rate of bone formation in vivo

Briefly, mice (P30) were injected (intraperitoneally) with fluorochrome-labeled calcein green (10 mg/kg, Sigma–Aldrich) twice (5 days interval). The mice were sacrificed 2 d after the second injection. The femurs were fixed in 70% (vol/vol) ethanol overnight, embedded in methyl methacrylate, and sectioned at 7–10 μm. Images were obtained using Zeiss LSM 800 fluorescence microscope. The mineral apposition rate (MAR) in μm/d and bone formation rate (BFR) [BFR  =  MAR × MS (mineral surface)/BS (bone surface)] were calculated from fluorochrome double labels at the endosteum surfaces.

### Measurements of serum levels of PYD

Blood samples were collected, allowed to clot for 30 minutes, and centrifuged for 10 minutes at 3000 rpm. Serum was frozen at −80 °C until use. PYD was measured in duplicate with MicroVue Serum PYD Elisa kit (Quidel Corporation) respectively, as described previously^65^. Concentrations were obtained by comparing readings against standard curves.

### Cell surface biotinylation and proteomic analysis

Cultured OBs (from the long bone of P90 male mice) were incubated with Sulfo-NHS-biotin (ThermoFisher Scientific) in PBS for 45 min at 4 °C, and treated with 10 mM glycine for 20 min at 4 °C to terminate the crosslinking reaction. After rinse with PBS, cells were lysed in RIPA buffer. Biotinylated proteins were precipitated overnight with avidin agarose beads (Pierce). Finally, beads were washed three times with PBS, and 1× SDS loading buffer was added to collect biotinylated surface proteins.

For proteomic assay, biotinylated surface proteins were separated by SDS-Page gel and submitted for LC-MS/MS analysis. The analysis was performed in the mass spectrometry laboratory at the Lerner Research Institute of the Cleveland Clinic Foundation. The LC-MS system was a ThermoScientific Orbitrap Elite mass spectrometer system. The HPLC column was a Dionex 15 cm × 75 μm id Acclaim Pepmap C18, 2 μm, 100 Å reversed- phase capillary chromatography column. 5 μL volumes of the extract were injected and the peptides eluted from the column by an acetonitrile/0.1% formic acid gradient at a flow rate of 0.3 μL/min were introduced into the source of the mass spectrometer on-line. The microelectrospray ion source is operated at 2.5 kV. The digest was analyzed using the data dependent multitask capability of the instrument acquiring full scan mass spectra to determine peptide molecular weights and product ion spectra to determine amino acid sequence in successive instrument scans. The data were analyzed by using all CID/HCD spectra collected in the experiment to search the mouse UniProtKB database with the search Sequest. The protein and peptide identifications were validated with the program scaffold.

### Collagen-degradative assays

The culture dishes were coated with type I collagen according to the instructions (A10483-01, Gibco). In brief, the rat tail Collagen I (3 mg/mL) were mixed with dH_2_O, 1 N NaOH, and 10× PBS to achieve at pH of 6.5–7.5. The mixed Collagen I were then dispensed into the desired dishes immediately and incubate at 37 °C in humidified incubator for 30–40 minutes or until a firm gel is formed. The dishes was rinsed with sterile 1× PBS and labeled with the fluorescent dye (Alexa fluor 488 or 594 NHS Ester) as described previously^42,67^. Primary cultured OBs or osteocytes were seeded on the fluorescently labeled collagen gel and protected from light in the incubator for 1–3 days. The fluorescence will disappear when the cells breakdown the surrounding collagen. Fluorescent images were obtained using Zeiss LSM 800 fluorescence microscope.

### Lentiviral particle injection in vivo

P15 mice were anesthetized by intraperitoneal injection of ketamine (75–100 mg/kg) and xylazine (5–10 mg/kg). Adequate depth of anesthesia was confirmed by the absence of response to toe pinch prior to surgical manipulation. The femoral region was shaved and aseptically prepared using iodine and ethanol solutions. A fine glass micropipette (tip diameter 20–40 μm) was carefully inserted into the interface between muscle and femur to deliver control or pLV-FLEX-MMP14-eGFP lentiviral particles(2–5 μL) onto the femoral surface. Following delivery, the pipette was slowly withdrawn after 4–5 minutes to minimize reflux of the viral suspension. Animals were maintained under anesthesia during the procedure and allowed to recover on a heating pad. Postoperative recovery was monitored until animals resumed normal activity. One month after lentiviral delivery, mice were euthanized as described above, and tissues were collected for analysis.

### RNA isolation and real time-PCR

Total RNA was isolated by Trizol extraction (Invitrogen, Carlsbad, CA, USA). Q-PCR was performed by using Quantitect SYBR Green PCR Kit (Bio-Rad) with a Real-Time PCR System (Opticon Monitor 3). *Mmp14* primers (5′-AGTGCCCTATGCCTACATCC-3′ and 5′-GGAACCCTCCTTCACCATCA-3′), *Atp6ap2* primers (5′-AGGAGATCGAATCCCAGAC-3’ and 5’-GTGCCAGTTTATCCACCC-3′), *Alp* primers (5′-GTAACCGCTGCCCGAATC-3’ and 5’-TCACTCCCACTGTGCCCTC-3′), *Sost* primers (5′-CCCGAACGGACCGGATTT-3’ and 5’-GCTTGCACTTGCACGAGGC-3′) and *Actb* (β-actin) primers (5′-AGGTCATCACTATTGGCAACGA-3′ and 5′-CATGGATGCCACAGGATTCC-3′) were used.

### Statistical analysis

For in vivo studies, 3–6 mice per genotype per assay were used. For in vitro cell biological and biochemical studies, each experiment was repeated at least three times. Statistical analyses were performed with GraphPad Prism 8 using unpaired student’s two-tailed *t* test, one-way ANOVA with Tukey post hoc test, or two-way ANOVA with Bonferroni post doc test. In all experiments, the significance level was set at *P* < 0.05 (*P < 0.05, **P < 0.01, ***P < 0.001).

### Reporting summary

Further information on research design is available in the Nature Portfolio Reporting Summary linked to this article.

## Supplementary information

Supplementary Information

Description of Additional Supplementary files

Supplementary Data 1

Reporting Summary

Transparent Peer Review file

## Data Availability

All data supporting the findings of this study are available within the Article and its Supplementary Information files. The source data are provided in Supplementary Data 1. Original western blot images are provided in Supplementary Fig. 11. The mass spectrometry proteomics data are publicly available through the ProteomeXchange Consortium via the PRIDE repository under accession number PXD079754 (DOI: 10.6019/PXD079754). Additional information is available from the corresponding author upon reasonable request.
