# Supplementary material for: ATP6AP2 in osteoblasts couples cortical bone matrix remodeling to osteocyte maturation via MMP14 activation in mice
Source: Commun Biol. 2026 Jul 11;9:1454. doi: 10.1038/s42003-026-10615-z (PMC13737131; doi:10.1038/s42003-026-10615-z)
Supplement: Supplementary file 1 [file 42003_2026_10615_MOESM1_ESM.pdf]

Supplementary Information to the paper

ATP6AP2 in osteoblasts couples cortical bone matrix remodeling to osteocyte maturation via MMP14 activation in mice

Lei Xiong[1], Hao-Han Guo[1], Jin-Xiu Pan[1], Xiao Ren[1], Daehoon Lee[1], Lin Mei[1], and Wen-Cheng Xiong[1,*]

[1] Department of Neurosciences, School of Medicine, Case Western Reserve University, Cleveland, OH 44106

*, Corresponding author
Dr. Wen-Cheng Xiong, Department of Neurosciences, School of Medicine, Case Western Reserve University, 10900 Euclid Ave, Cleveland, Ohio, 44106
Email: Wen-Cheng.Xiong@case.edu
Phone: +1 216-368-4865

Running title: ATP6AP2 promoting cortical bone matrix modeling and osteocyte distribution and maturation

Keywords: ATP6AP2, MMP14, Osteoblasts, Osteocytes

Supplementary Figures

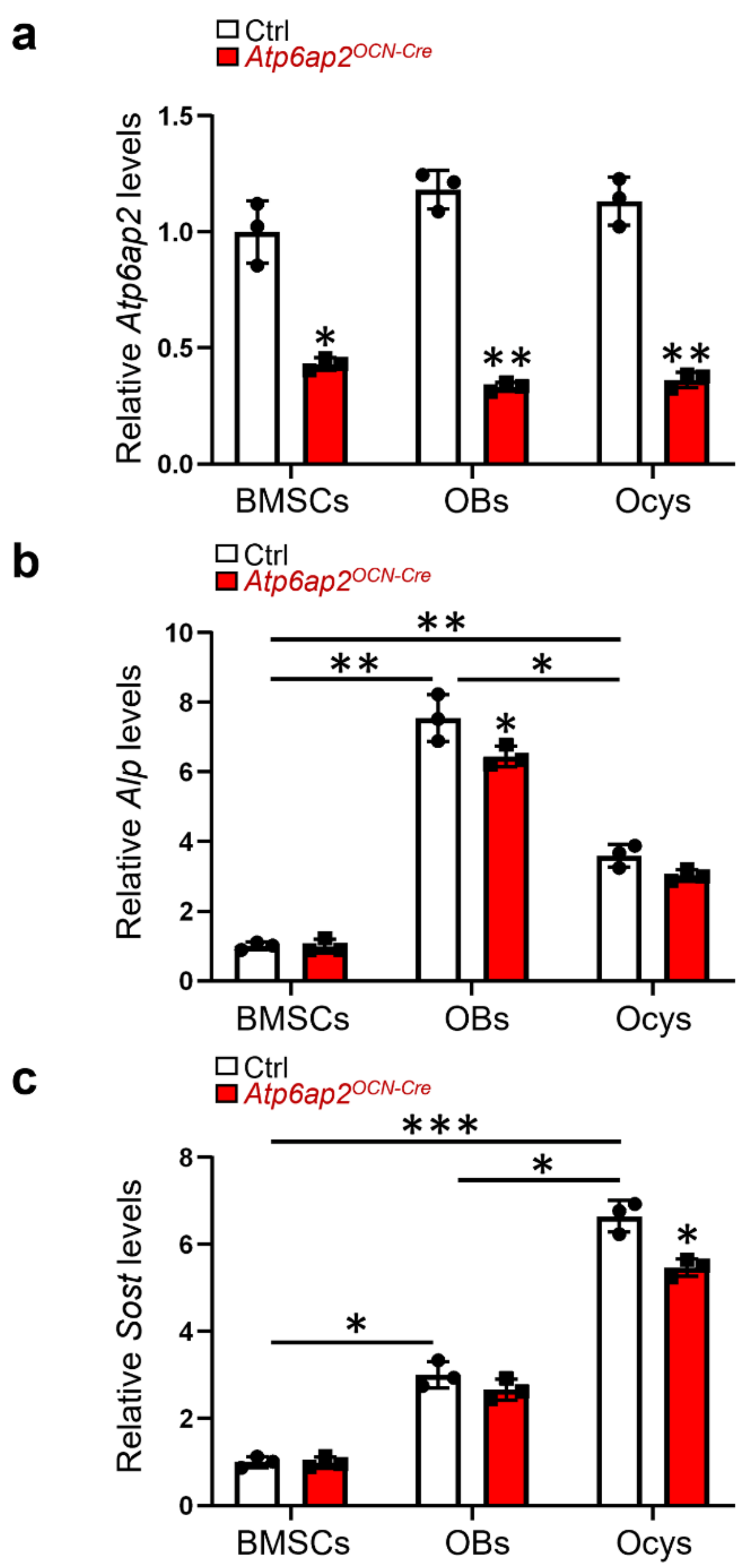

Figure S1. Gene expression in primary cultured cells.

(a-c) Q-PCR analysis of *Atp6ap2*, Alkaline phosphatase (*Alp*, an early marker of osteoblast differentiation) and *Sost* (a marker of osteocytes) expression in BMSCs, osteoblasts, and osteocytes derived from P90 male control and *Atp6ap2*$^{OCN\text{-}Cre}$ mice.

Data in (A) are presented as mean± SD (n = 3). *, P< 0.05, **, P< 0.01, ***, P< 0.001 as determined by two-way ANOVA followed by Bonferroni's post hoc test.

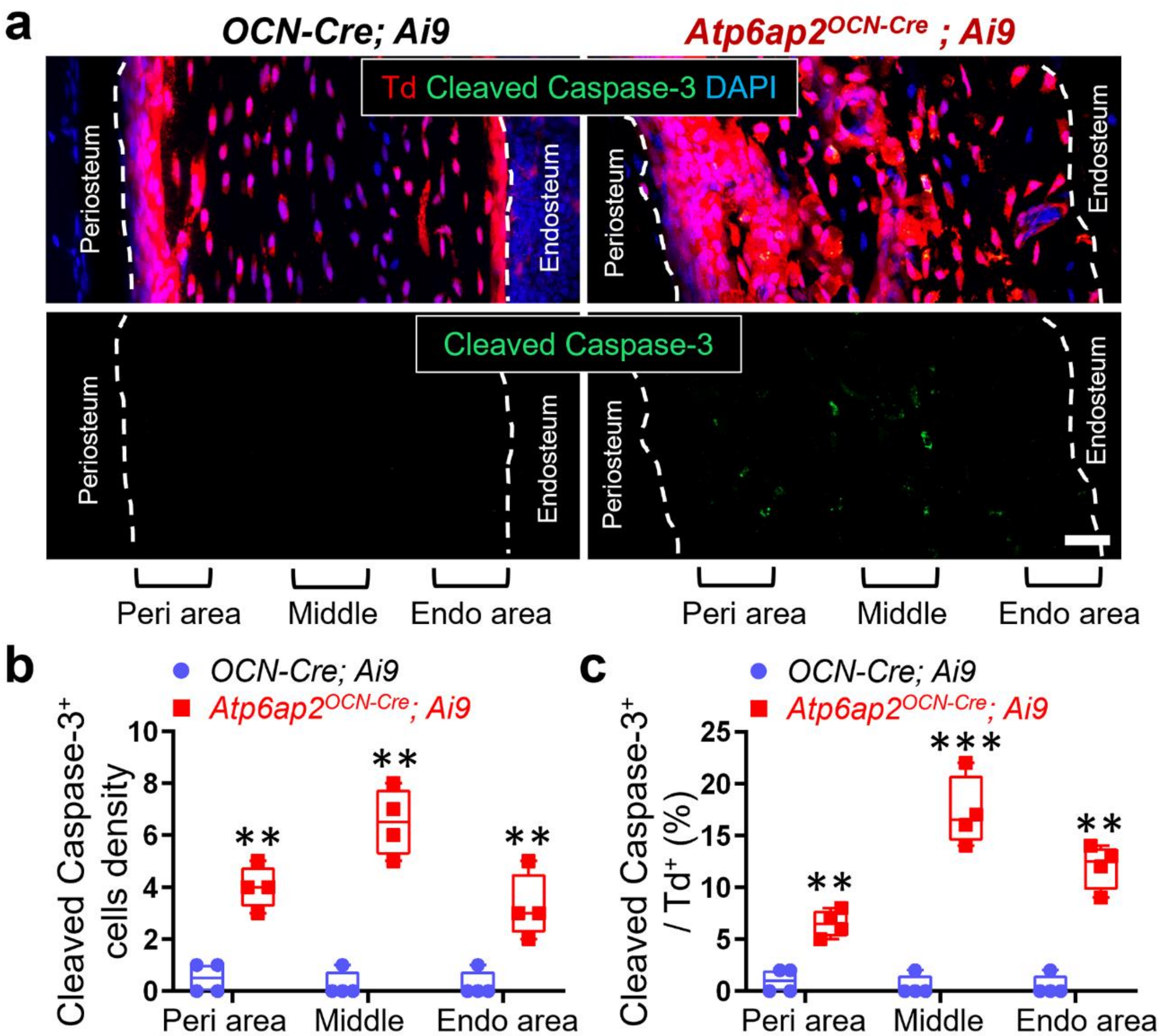

Figure S2. Increased cell death in the cortical bone of *Atp6ap2$^{OCN\text{-}Cre}$* mice

(a-c) Immunofluorescence of femur cortical bone in P30 *OCN-Cre; Ai9* and *Atp6ap2$^{OCN\text{-}Cre}$; Ai9* mice by using Cleaved caspase-3 (cell apoptosis marker) antibody. Bar, 20 µm. Quantification analysis was shown in b and c.

Data in (b) and (c) are shown as box plots with individual data points. The center line indicates the median, the box indicates the interquartile range, and whiskers indicate minimum to maximum values (n=4 animals per genotype). P values obtained by unpaired two-tailed t-test. **, P< 0.01. ***, P< 0.001.

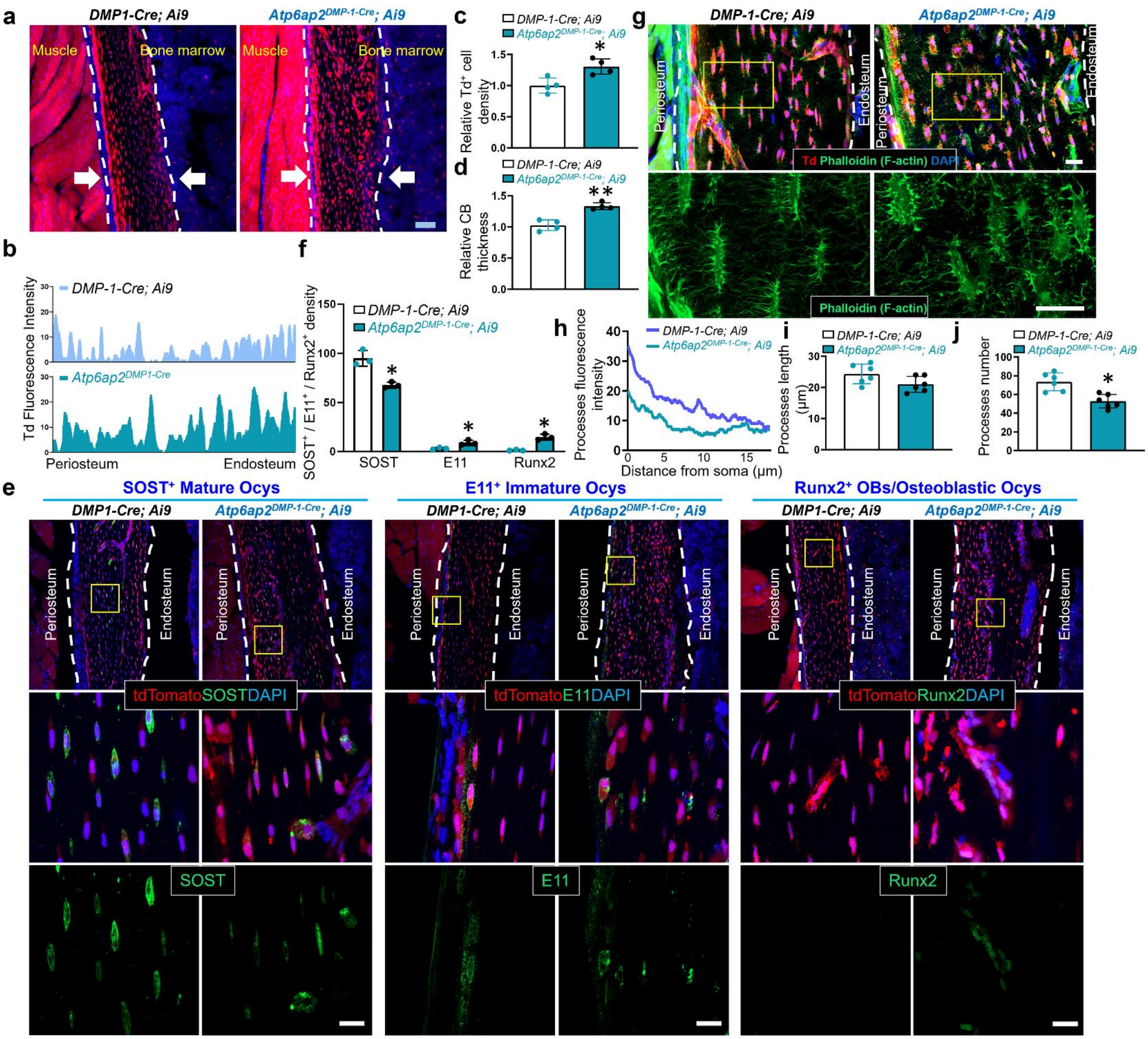

Figure S3. Similar, but weaker, bone deficit in *Atp6ap2*$^{\text{DMP-1-Cre}}$ mice.

(a) Td-Tomato expression in the cortical bone region (diaphysis) of femur sections from P30 male mice with targeted deletion of ATP6AP2 in cells expressing Dmp-1 (TdTomato^{+}, *Atp6ap2$^{Dmp\text{-}1\text{-}Cre}$; Ai9*) and their littermates (*Dmp-1-Cre; Ai9*). Bar, 50 µm.

(b-d) Quantifications of the TdTomato red fluorescence intensity (b), the tdTomato^{+} cell density (c) and cortical bone thickness (d).

(e) Immunofluorescence of femur cortical bone (diaphysis) using SOST (mature osteocytes marker), E11 (early, immature osteocyte marker) and Runx2 (preosteoblast, osteoblast, osteoblastic osteocyte marker) antibodies. Bar, 20 μm.

(f) Quantification of the $SOST^{+}$, $E11^{+}$ and $Runx2^{+}$ cells density.

(g) Immunofluorescence labeling of osteocytes processes with phalloidin-488 for f-actin (green) in femur cortical bone (diaphysis) of P30 male *DMP-1-Cre; Ai9* and *Atp6ap2*$^{DMP\text{-}1\text{-}Cre}$*; Ai9* mice. Bar, 20 μm

(h-j) Quantification of processes fluorescence intensity (h), processes length (i) and processes number per osteocyte (j).

Data in (c), (d), (f), (i) and (j) are presented as mean± SD (n = 3, 4 or 6 animals per genotype). *, $P< 0.05$, **, $P< 0.01$ as determined by unpaired two-tailed t-test.

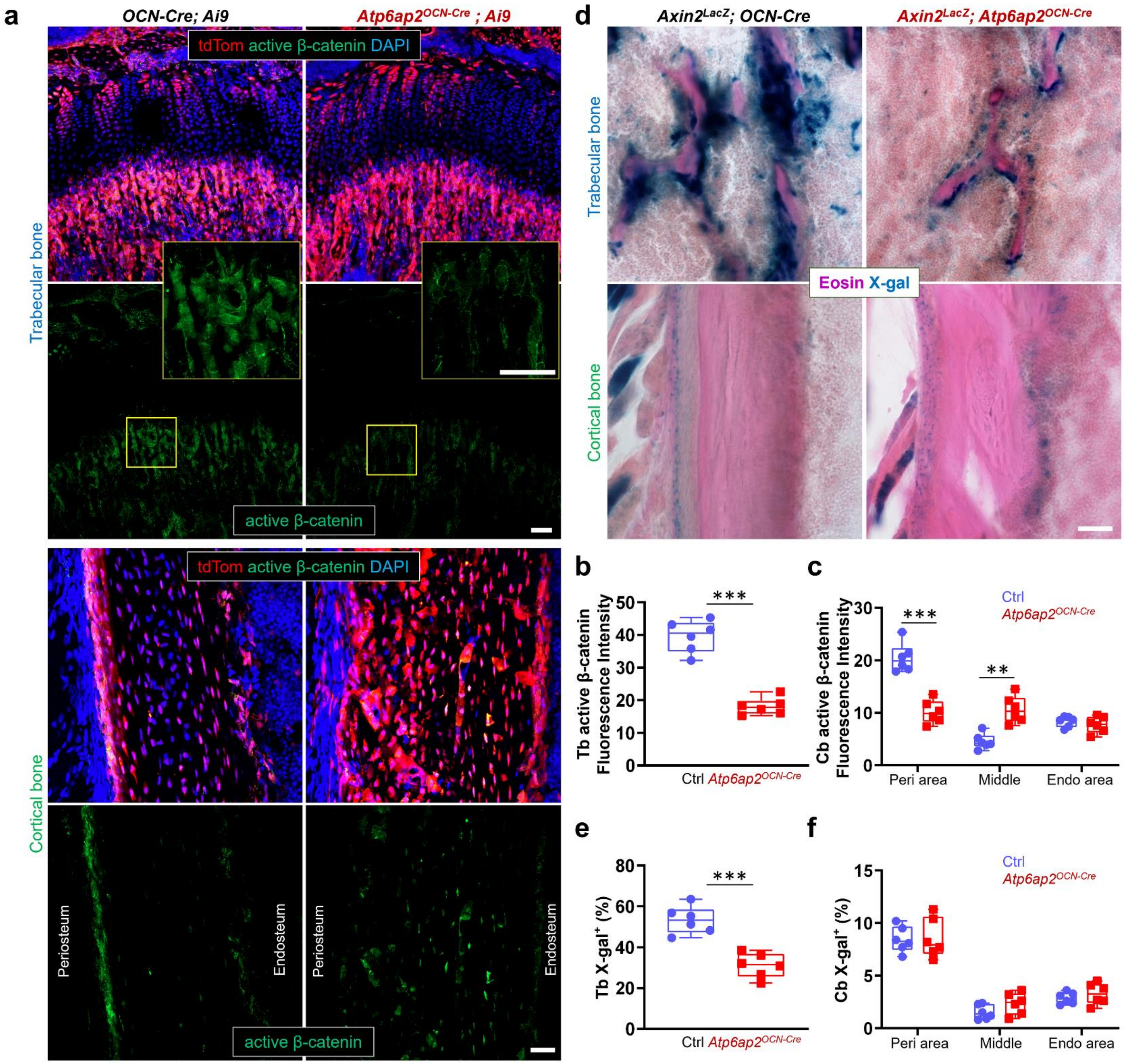

Figure S4. *Atp6ap2*-cKO reduces Wnt/β-Catenin signaling activity in trabecular bone but not in cortical bone

(a-c) Immunofluorescence of femur cortical bone in P30 *OCN-Cre; Ai9* and *Atp6ap2*$^{OCN\text{-}Cre}$*; Ai9* mice by using active β-catenin antibody. Bar, 20 µm. Quantification analysis was shown in b and c.

(d-f) Selectively decreased β-catenin signaling in the trabecular bone of *Atp6ap2*$^{OCN\text{-}Cre}$ mice at P30, by Axin2^{LacZ} reporter activity. Ctrl and *Atp6ap2*$^{OCN\text{-}Cre}$ mice are crossed with *Axin2*^{LacZ} reporter line and β-galactosidase activity is revealed by X-gal staining. Representative images were shown in d. Bar, 50 μm. Quantification analysis were shown in e and f.

Data in (b-c) and (e-f) are shown as box plots together with individual data points, and whiskers indicate minimum to maximum (n=6 animals per genotype). P values obtained by unpaired two-tailed t-test. **, P< 0.01. ***, P< 0.001.

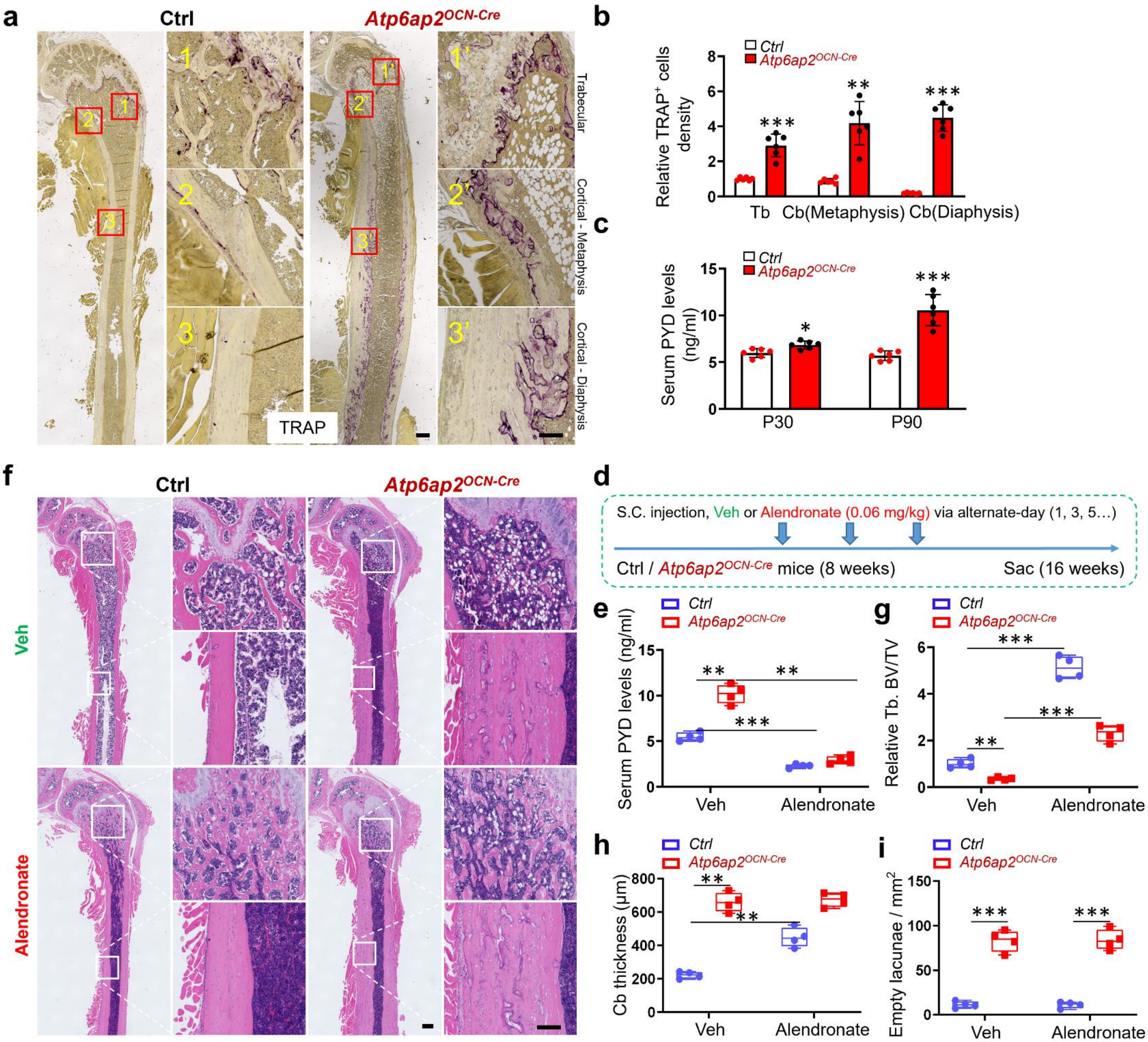

Figure S5. Increased bone resorption that contributes to the decreased trabecular bone-mass, but not cortical bone-mass, in *Atp6ap2*$^{OCN\text{-}Cre}$ mice.

(a) TRAP staining analysis of femur sections from P90 male ctrl and *Atp6ap2*$^{OCN\text{-}Cre}$ mice. Bar, 300 μm in the main images and 100 μm in the boxed enlarged views.

(b) Quantification analysis of $TRAP^+$ cell number per unit bone surface area.

(c) ELISA analysis of serum pyridinoline (PYD) levels.

(d) Experimental strategy. 8 weeks old male ctrl and $Atp6ap2^{OCN\text{-}Cre}$ mice were administered by subcutaneous injection of 0.06 mg/kg alendronate or vehicle (0.9% sodium chloride) via alternate day for 8 weeks. The serum and femur samples collected after treatments were subjected to ELISA analysis and H&E staining.

(e) ELISA analysis of serum pyridinoline (PYD) levels.

(f) Representative images of H&E stained femur sections from indicted groups. Bar, 300 µm in the main images and 100 µm in the boxed enlarged views.

(g-i) Quantification of trabecular bone (Tb) volumes over total volumes (BV/TV), cortical bone thickness and empty lacunae.

The values of mean ± SD (n = 6 animals per genotype) were shown in (b) and (c). Data in (e) and (g-i) are shown as box plots together with individual data points, and whiskers indicate minimum to maximum (n=4 animals per genotype). P values obtained by unpaired two-tailed t-test or two-way ANOVA with Bonferroni post hoc analysis for multiple comparisons test. *, P < 0.05. **, P< 0.01. ***, P < 0.001.

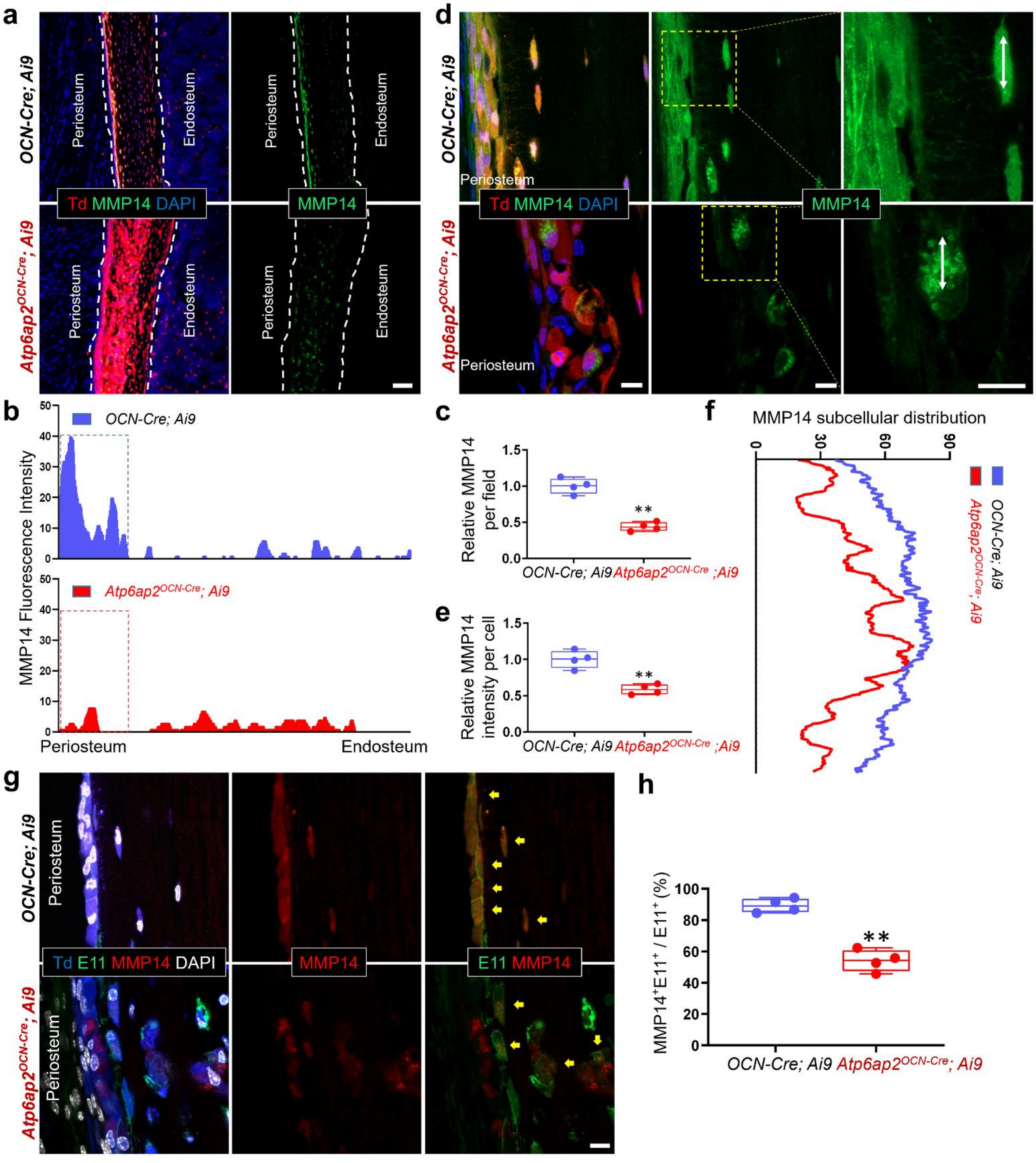

Figure S6. Reduced MMP14 surface distribution in *Atp6ap2*-KO osteocytes.

(a-c) Immunofluorescence of femur cortical bone (diaphysis) in P30 *OCN-Cre; Ai9* and *Atp6ap2$^{OCN\text{-}Cre}$; Ai9* mice using MMP14 antibody. Representative images were shown in a. Bar,

50 μm. Quantification analyses of MMP14 fluorescence intensity was shown in b and c.

(d-f) The vesicle-like distribution of MMP14 in *Atp6ap2*-KO cells. Immunostaining images of MMP14 in the periosteum area of P30 *OCN-Cre; Ai9* and *Atp6ap2*$^{OCN\text{-}Cre}$*; Ai9* mice were shown in d. Bar, 20 μm. Quantification analyses of MMP14 fluorescence intensity per cells and fluorescence distribution in the marked cells were shown in e and f.

(g, h) MMP14 high expressed in immature osteocytes. Immunofluorescence images of femur cortical bone in P30 *OCN-Cre; Ai9* and *Atp6ap2*$^{OCN\text{-}Cre}$*; Ai9* mice using MMP14 and E11(gp38) antibodies were shown in g. Bar, 20 μm. Quantification analysis of the ratio of MMP14^{+}; E11^{+} over total E11^{+} cells were shown in h.

Data in (c), (e) and (h) are shown as box plots together with individual data points, and whiskers indicate minimum to maximum (n = 4 animals per genotype). **, $P< 0.01$ as determined by unpaired two-tailed t-test.

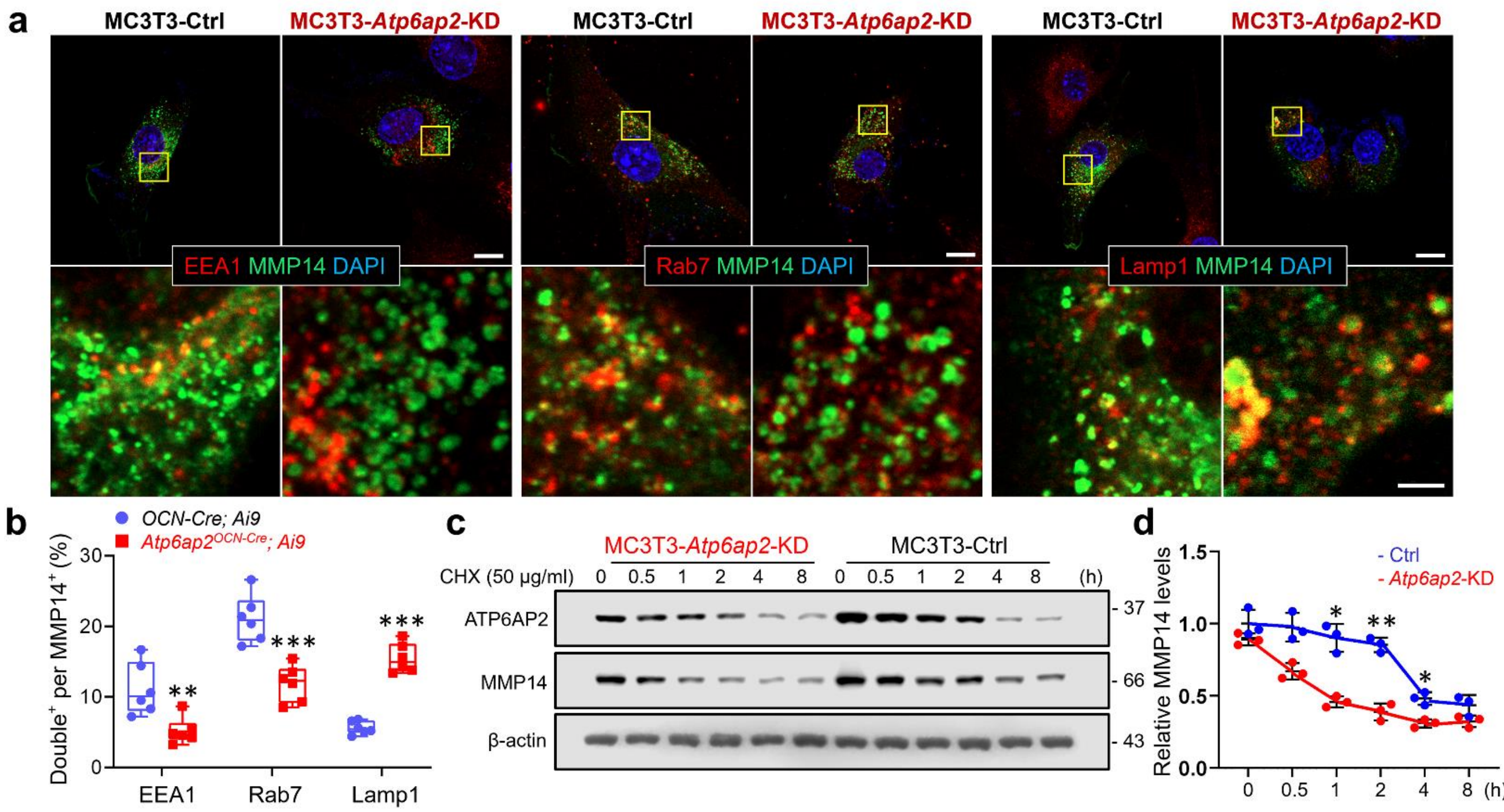

Figure S7. ATP6AP2 regulates MMP14 protein stability.

(a-b) Co-immunostaining analysis of MMP14 with EEA1 (a marker for early endosomes), Rab7 (a marker for late endosomes) or Lamp1 (a marker for late endosomes and early lysosomes) in control and *Atp6ap2*-KD MC3T3 cells. Representative images were shown in A. Images marked with yellow squares were amplified and shown in the bottom. Bar, 10 μm (single cell) and 2 μm (zoom). The co-localization index of MMP14 with indicated markers (EEA1, Rab7 and LAMP1) was determined by the measurement of overlapped signaling (yellow fluorescence) over total MMP14 signal.

(c-d) Time-course analysis of MMP14 protein levels after cycloheximide (CHX) treatment. Ctrl or *Atp6ap2*-KD MC3T3 cell lines were treated with 50 μg/ml CHX for the indicated time. ATP6AP2 and MMP14 protein levels were analyzed by Western blotting. Representative blots are shown in c, and quantification analysis is presented in d.

Data in (b) are shown as box plots together with individual data points, and whiskers indicate minimum to maximum (n=6 independent experiments). Data in (d) are shown as mean ± SD from three separate experiments. *, $P < 0.05$, **, $P < 0.01$, ***, $P < 0.001$ as determined by unpaired two-tailed t-test or one-way ANOVA followed by Tukey's post hoc test.

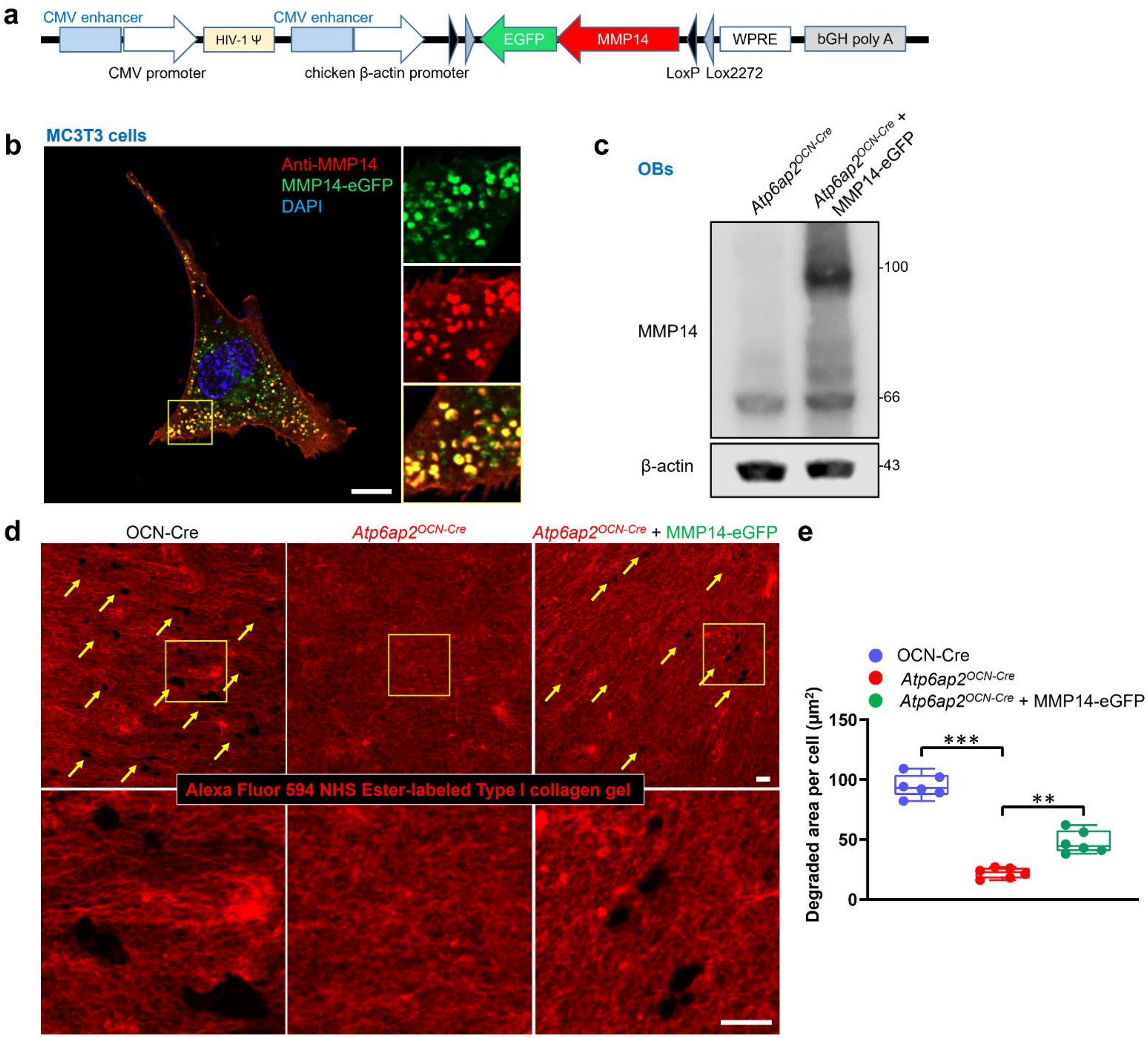

Figure S8. Overexpression of MMP14 promotes collagen-degradative activity of *Atp6ap2*-KO OBs

(a) Generation of MMP14-eGFP lentiviral particles.

(b) Immunofluorescence analysis of MMP14-eGFP lentivirus plasmid expression in MC3T3 cells. Cells were co-transfected with MMP14-eGFP lentivirus and cre plasmids. Bar, 10 µm.

(c) Western blot analysis of MMP14-eGFP, which was increased in *Atp6ap2*$^{OCN\text{-}Cre}$ + MMP14-eGFP OBs. OBs were infected with lentivirus and then purified by FACS.

(d) Confocal micrographs showing degradation of the fluorescent type I collagen layer, indicated by dark holes, by ctrl, *Atp6ap2*-KO and *Atp6ap2*-KO with MMP14-eGFP overexpression OBs. The type I collagen gel was labeled by Alexa fluor 594 NHS ester dye before seeding the cells. Bar, 10 μm.

(e) Quantitative measurement in d. Data are shown as box plots together with individual data points, and whiskers indicate minimum to maximum (n=6 independent experiments). P values obtained by one-way ANOVA followed by Tukey post hoc test. **, P< 0.01. ***, P< 0.001.

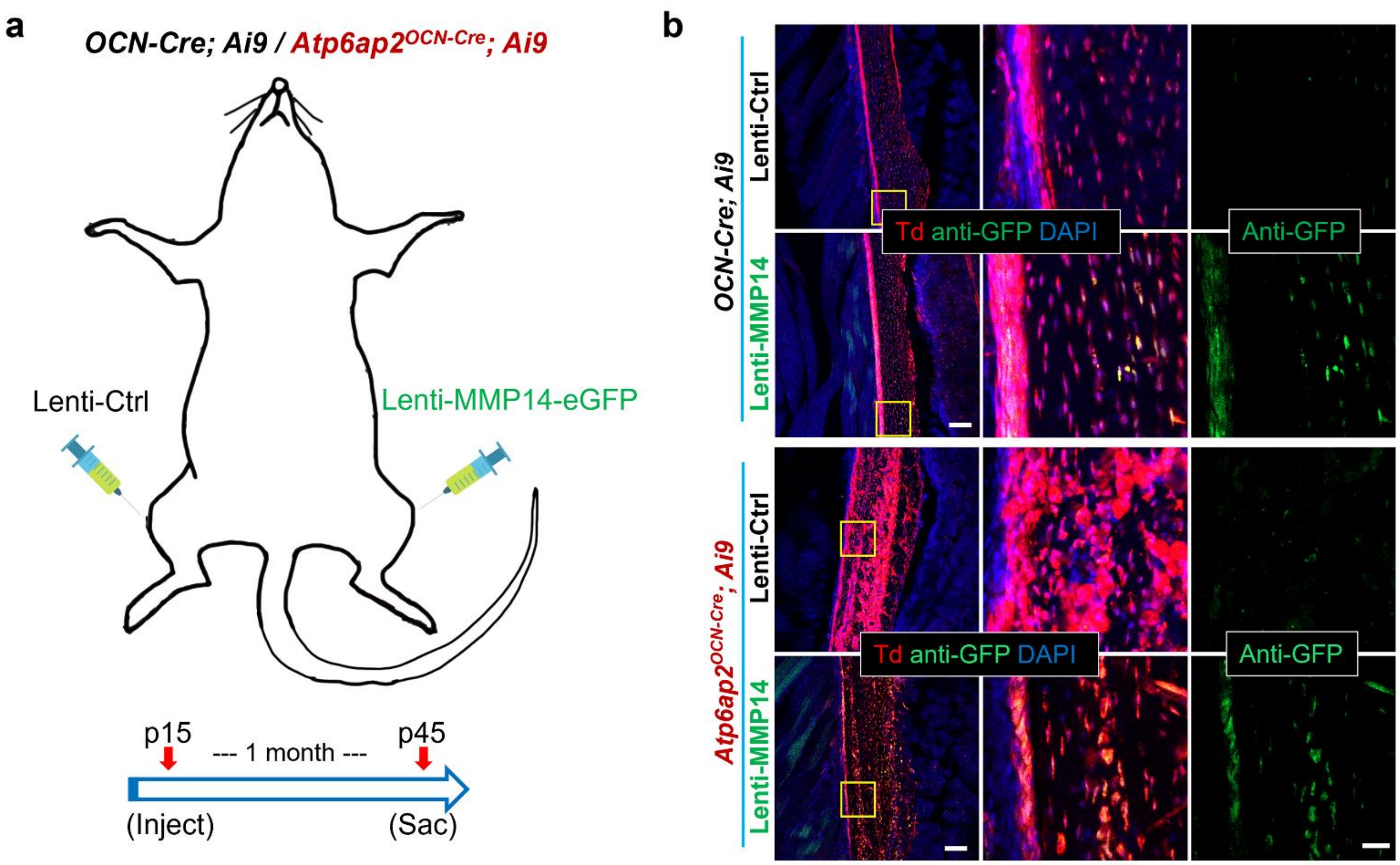

Figure S9. Expression of MMP14-eGFP in femur of mice.

(a) Ctrl or MMP14-eGFP lentiviral particles were injected into the surface of femur of male *OCN-Cre; Ai9* or *Atp6ap2*$^{OCN\text{-}Cre}$; *Ai9* mice at the age of P15. In order to compare the effect of MMP14 overexpression, ctrl or MMP14-eGFP lentiviral particles were injected into different hind legs of the same mouse. Samples were collected 1 month after injection.

(b) Immunofluorescence analysis of MMP14-eGFP expression in femur of indicted mice. Bar, 100 μm in the main images and 20 μm in the boxed enlarged views.

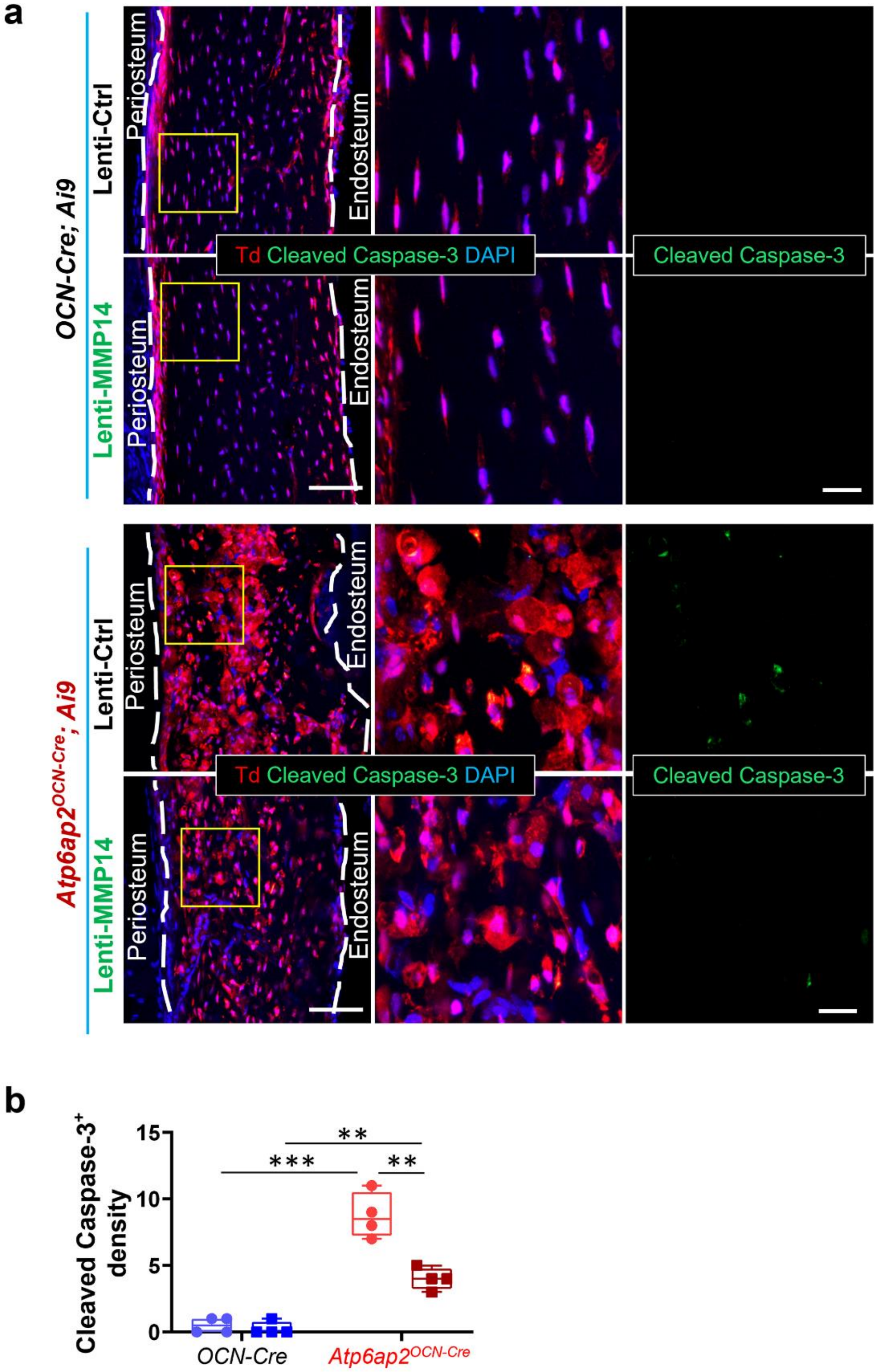

Figure S10. MMP14 attenuation of the cortical osteocyte deficits in *Atp6ap2$^{OCN\text{-}Cre}$* mice.

(a-b) Immunofluorescence labeling of osteocytes with cleaved caspase-3 in femur cortical bone (diaphysis) of P45 *OCN-Cre; Ai9* and *Atp6ap2$^{OCN\text{-}Cre}$; Ai9* mice with ctrl or MMP14-eGFP

expression. Bar, 80 μm in the main images and 20 μm in the boxed enlarged views. Quantification were shown in b.

Data in (b) is shown as box plots together with individual data points, and whiskers indicate minimum to maximum (n=4 animals). P values obtained by two-way ANOVA followed by Bonferroni's post hoc test. **, P< 0.01. ***, P< 0.001.

Fig. 5e

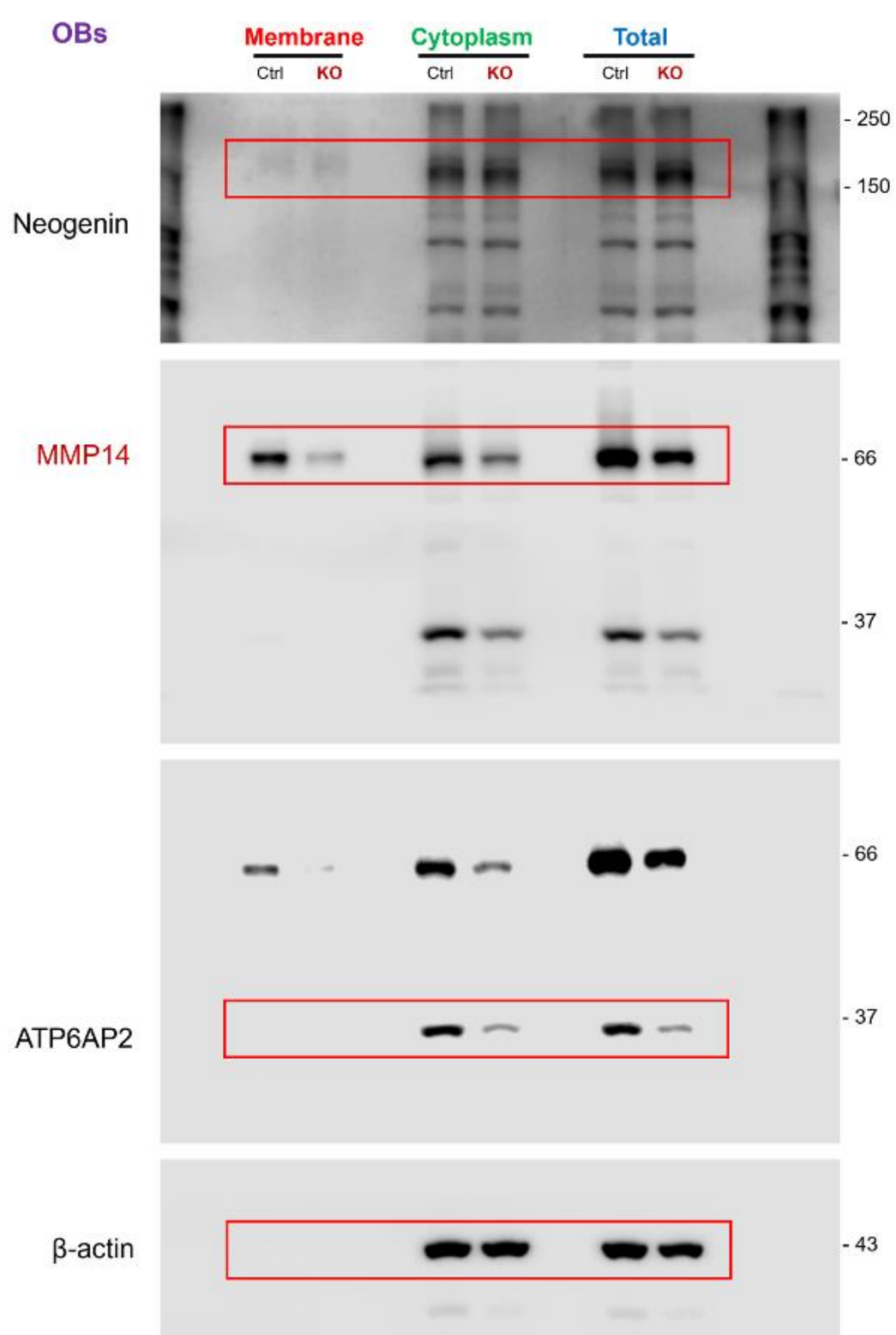

Fig. 6b

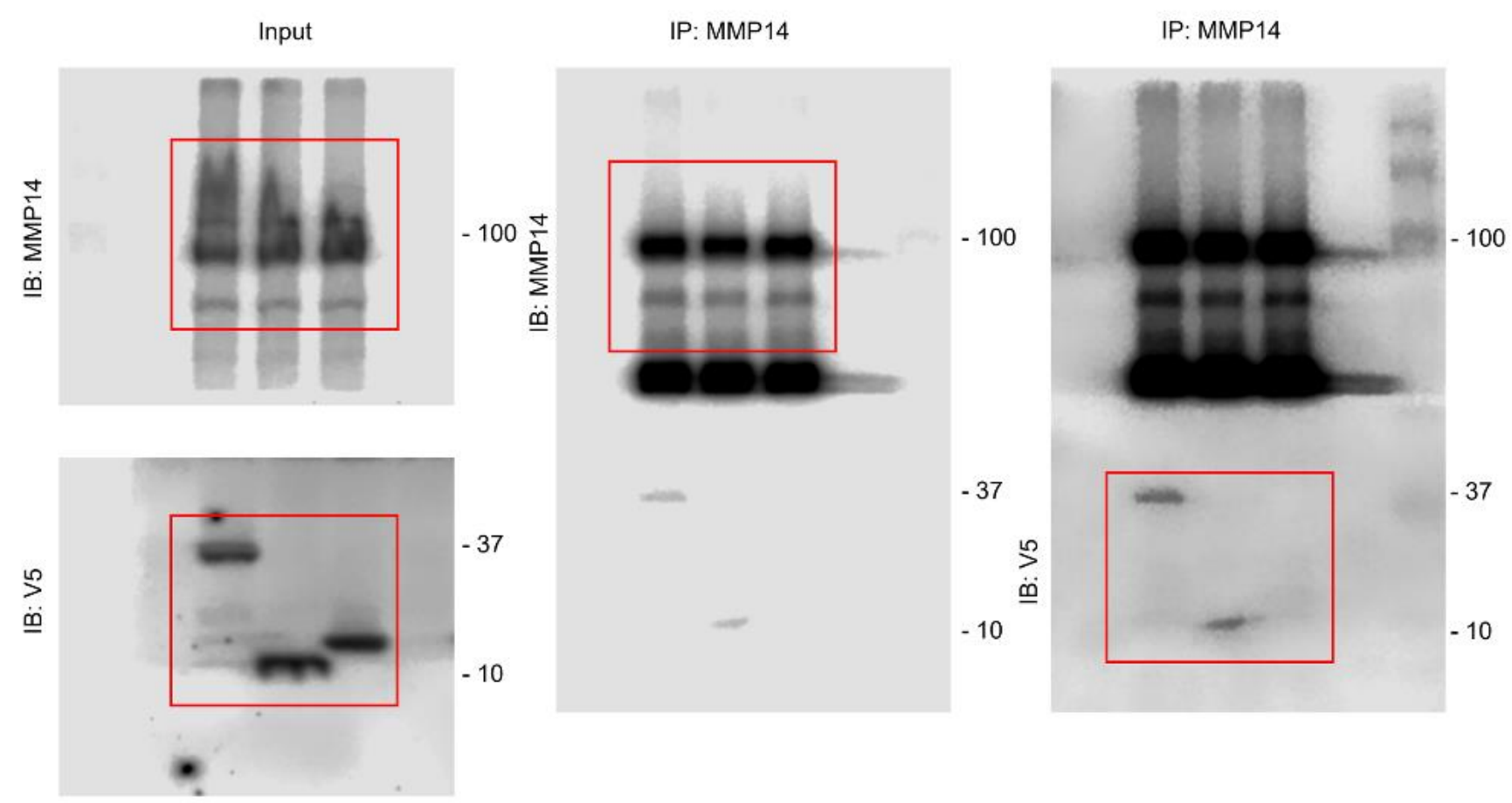

Fig. S7c

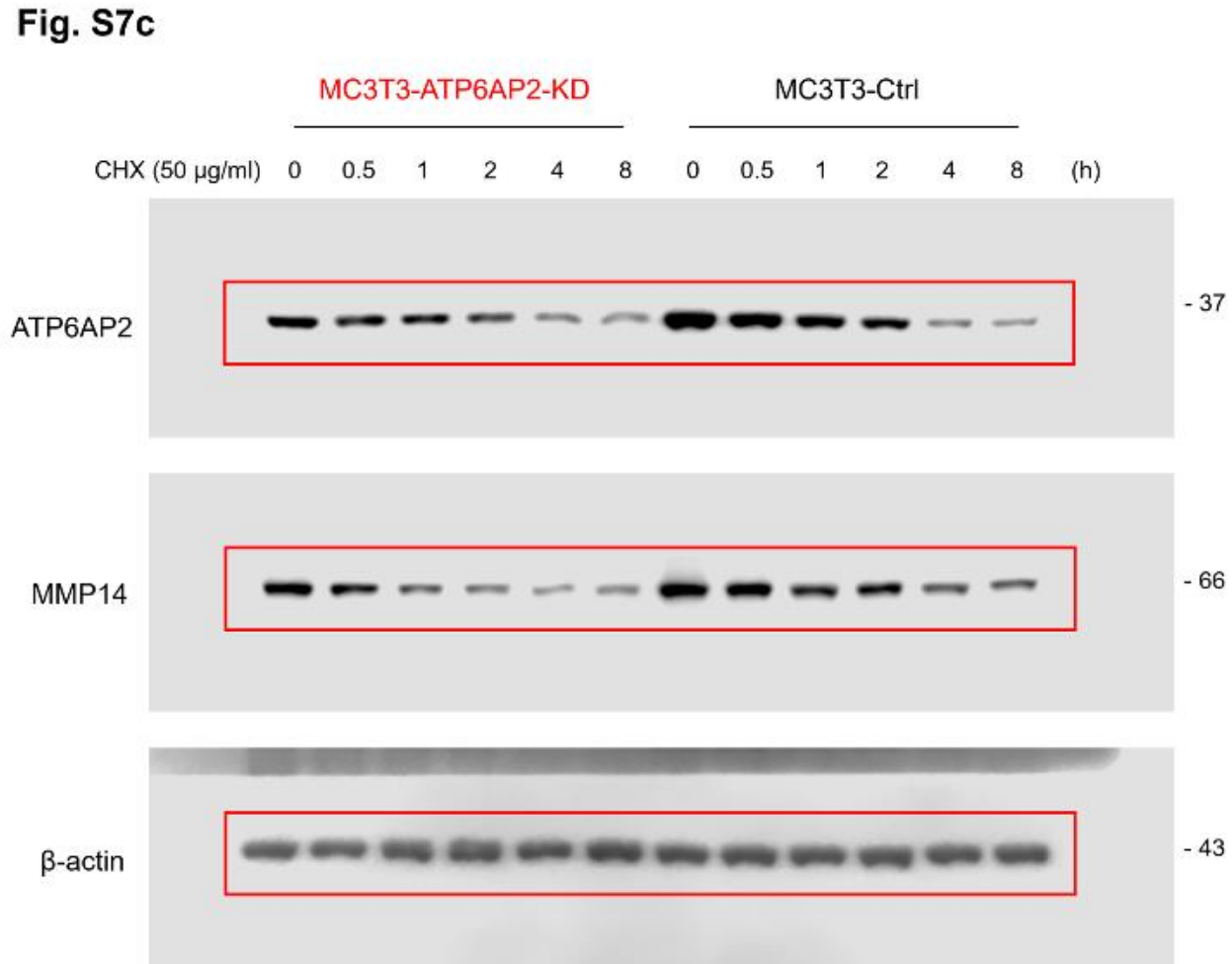

Fig. S8c

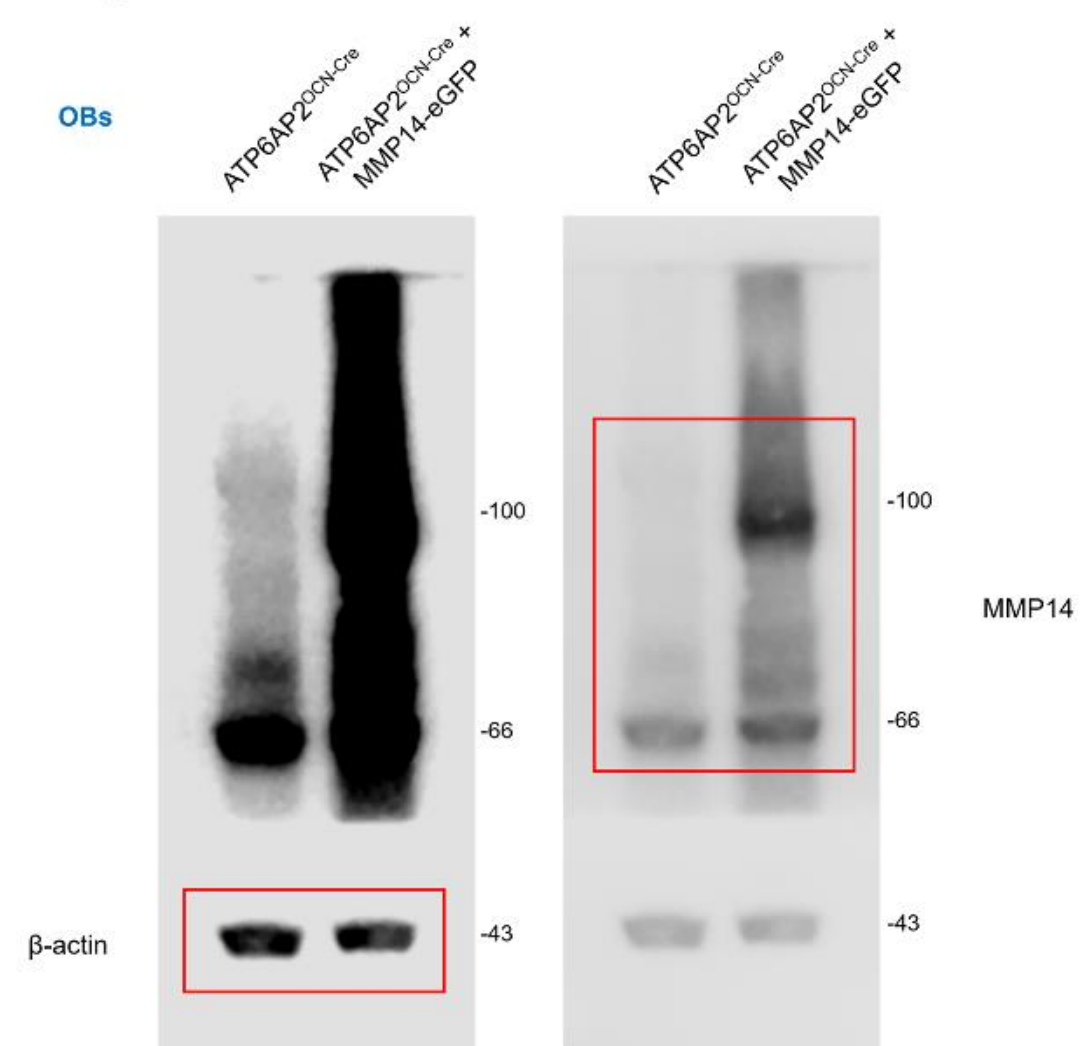

Figure S11. Uncropped western blot images.

Uncropped and unprocessed western blot images corresponding to Fig. 5e, Fig. 6b, Fig. S7c and Fig. S8c are provided. Red boxes indicate the regions shown in the main figures.
