# Supplementary material for: ATP6AP2 in osteoblasts couples cortical bone matrix remodeling to osteocyte maturation via MMP14 activation in mice
Source: Commun Biol. 2026 Jul 11;9:1454. doi: 10.1038/s42003-026-10615-z (PMC13737131; doi:10.1038/s42003-026-10615-z)
Supplement: Supplementary file 2 [file 42003_2026_10615_MOESM2_ESM.pdf]

Description of Additional Supplementary files

File name: Supplementary Data 1

Description: Source data are provided as Excel file in Supplementary Data 1
