# Supplementary material for: ATP6AP2 in osteoblasts couples cortical bone matrix remodeling to osteocyte maturation via MMP14 activation in mice
Source: Commun Biol. 2026 Jul 11;9:1454. doi: 10.1038/s42003-026-10615-z (PMC13737131; doi:10.1038/s42003-026-10615-z)
Supplement: Supplementary file 3 [file 42003_2026_10615_MOESM4_ESM.pdf]

nature portfolio

Corresponding author(s): Wen-Cheng Xiong

Last updated by author(s): Jun 22, 2026

Reporting Summary

Nature Portfolio wishes to improve the reproducibility of the work that we publish. This form provides structure for consistency and transparency in reporting. For further information on Nature Portfolio policies, see our Editorial Policies and the Editorial Policy Checklist.

Statistics

For all statistical analyses, confirm that the following items are present in the figure legend, table legend, main text, or Methods section.

n/a	Confirmed	
☐	☒	The exact sample size (*n*) for each experimental group/condition, given as a discrete number and unit of measurement
☐	☒	A statement on whether measurements were taken from distinct samples or whether the same sample was measured repeatedly
☐	☒	The statistical test(s) used AND whether they are one- or two-sided<br>*Only common tests should be described solely by name; describe more complex techniques in the Methods section.*
☐	☒	A description of all covariates tested
☐	☒	A description of any assumptions or corrections, such as tests of normality and adjustment for multiple comparisons
☐	☒	A full description of the statistical parameters including central tendency (e.g. means) or other basic estimates (e.g. regression coefficient) AND variation (e.g. standard deviation) or associated estimates of uncertainty (e.g. confidence intervals)
☒	☐	For null hypothesis testing, the test statistic (e.g. *F*, *t*, *r*) with confidence intervals, effect sizes, degrees of freedom and *P* value noted<br>*Give P values as exact values whenever suitable.*
☒	☐	For Bayesian analysis, information on the choice of priors and Markov chain Monte Carlo settings
☒	☐	For hierarchical and complex designs, identification of the appropriate level for tests and full reporting of outcomes
☒	☐	Estimates of effect sizes (e.g. Cohen's *d*, Pearson's *r*), indicating how they were calculated

Our web collection on statistics for biologists contains articles on many of the points above.

Software and code

Policy information about availability of computer code

Data collection	ZEN (Zeiss) and ImageJ were used for fluorescence image processing. Confocal images were acquired using ZEN (Zeiss LSM 800) software. Histomorphometric images were acquired using KEYENCE BZ-X710 microscope software. Micro-CT were acquired using the Siemens Inveon Micro PET/CT scanner (10-µm resolution) or Mediso nanoScan SPECT/CT/PET system. For proteomic analysis, Cultured OBs were incubated with Sulfo-NHS-biotin and treated with glycine to terminate the crosslinking reaction. After rinse with PBS, cells were lysed in RIPA buffer. Biotinylated proteins were precipitated overnight with avidin agarose beads. The biotinylated surface proteins were collected and sent to the Lerner Research Institute of the Cleveland Clinic Foundation for mass spectrometry analysis.
Data analysis	ZEN (Zeiss), ImageJ and KEYENCE BZ-X710 software were used for image processing and data analysis. Avizo 3D software was used for the 3D reconstruction of micro-CT images and data analysis. For proteomic assay, biotinylated surface proteins were separated by SDS-Page gel and submitted for LC-MS/MS analysis. The analysis was performed in the mass spectrometry laboratory at the Lerner Research Institute of the Cleveland Clinic Foundation. The digest was analyzed using the data dependent multitask capability of the instrument acquiring full scan mass spectra to determine peptide molecular weights and product ion spectra to determine amino acid sequence in successive instrument scans. The data were analyzed by using all CID/HCD spectra collected in the experiment to search the mouse UniProtKB database with the search Sequest. The protein and peptide identifications were validated with the program scaffold. Most statistical analyses were performed with GraphPad Prism 8 using unpaired student's two-tailed t-test, one-way ANOVA with Tukey post hoc test, or two-way ANOVA with Bonferroni post doc test.

For manuscripts utilizing custom algorithms or software that are central to the research but not yet described in published literature, software must be made available to editors and reviewers. We strongly encourage code deposition in a community repository (e.g. GitHub). See the Nature Portfolio guidelines for submitting code & software for further information.

Data

Policy information about availability of data

All manuscripts must include a data availability statement. This statement should provide the following information, where applicable:

- Accession codes, unique identifiers, or web links for publicly available datasets
- A description of any restrictions on data availability
- For clinical datasets or third party data, please ensure that the statement adheres to our policy

Provide your data availability statement here.

Research involving human participants, their data, or biological material

Policy information about studies with human participants or human data. See also policy information about sex, gender (identity/presentation), and sexual orientation and race, ethnicity and racism.

Reporting on sex and gender	N/A
Reporting on race, ethnicity, or other socially relevant groupings	N/A
Population characteristics	N/A
Recruitment	N/A
Ethics oversight	N/A

Note that full information on the approval of the study protocol must also be provided in the manuscript.

Field-specific reporting

Please select the one below that is the best fit for your research. If you are not sure, read the appropriate sections before making your selection.

☒ Life sciences ☐ Behavioural & social sciences ☐ Ecological, evolutionary & environmental sciences

For a reference copy of the document with all sections, see nature.com/documents/nr-reporting-summary-flat.pdf

Life sciences study design

All studies must disclose on these points even when the disclosure is negative.

Sample size	No statistical methods were used to predetermine sample size. Sample sizes were chosen based on previous studies in the field and were sufficient to detect the reported effects.
Data exclusions	No data were excluded from the analyses.
Replication	All experiments were independently replicated with similar results. The number of biological replicates (n) is indicated in the figure legends.
Randomization	No randomization was performed because experimental groups (e.g., control and mutant mice) were defined by genotype. All animals within each group were processed using identical procedures to minimize potential bias.
Blinding	Blinding was not performed because experimental groups could be visually distinguished by genotype or fluorophore expression during imaging. All analyses were performed using standardized procedures to minimize potential bias.

Reporting for specific materials, systems and methods

We require information from authors about some types of materials, experimental systems and methods used in many studies. Here, indicate whether each material, system or method listed is relevant to your study. If you are not sure if a list item applies to your research, read the appropriate section before selecting a response.

Materials & experimental systems

n/a	Involved in the study	
☐	☒	Antibodies
☐	☒	Eukaryotic cell lines
☒	☐	Palaeontology and archaeology
☐	☒	Animals and other organisms
☒	☐	Clinical data
☒	☐	Dual use research of concern
☒	☐	Plants

Methods

n/a	Involved in the study	
☒	☐	ChIP-seq
☒	☐	Flow cytometry
☒	☐	MRI-based neuroimaging

Antibodies

Antibodies used

Mouse monoclonal antibodies, including β-actin (A1978, Sigma, 1:5000 dilution), Rab7 (ab50533, Abcam, 1:1000 dilution) and V5 (V8012, Sigma-Aldrich, 1:5000 dilution for WB, 1:500 dilution for IF), were used. Rabbit monoclonal antibodies Ki67 (ab16667, Abcam, 1:500 dilution), Active beta-Catenin (Ser33/37/Thr41 non-phospho, Cell signaling, 8814S, 1:200 dilution), MMP14 (ab51074, Abcam, 1:2000 dilution for WB, 1:400 dilution for IF) and Runx2 (ab192256, Abcam, 1:400 dilution) were used. Rabbit polyclonal antibody Cleaved Caspase-3 (9661, Cell signaling technology, 1:300 dilution), ATP6AP2 (HPA003156, Sigma Aldrich, 1:2000 dilution), neogenin (1:1000 for blotting), GFP (Clontech, 8367-1, 1:200 dilution) and EEA1 (ab2900, Abcam, 1:1000 dilution) were used. Goat SOST (AF1589, R&D, 1:200 dilution) antibody, Rat Lamp1 (1D4B-c, DSHB, 1:1000 dilution) antibody and Syrian hamster monoclonal E11(gp38 or gp36, ab11936, Abcam, 1:200 dilution) antibody were used. Alexa Fluor 488 phalloidin (f-actin, A12379, 5 assays/mL) was purchased from Thermo Fisher. Secondary antibodies were purchased from Jackson ImmunoResearch Laboratories, Inc.

Validation

All primary antibodies used in this study are commercially available and have been validated by the manufacturers for use in immunofluorescence, immunoprecipitation, or Western blotting in mouse tissues. Validation details are available on the respective suppliers' websites.

Eukaryotic cell lines

Policy information about cell lines and Sex and Gender in Research

Cell line source(s)

MC3T3-E1 and HEK293 cells were derived from ATCC.

Authentication

Their morphology and growth characteristics were consistent with published descriptions.

Mycoplasma contamination

MC3T3-E1 and HEK293 cells were tested negative for mycoplasma.

Commonly misidentified lines
(See ICLAC register)

No commonly misidentified cell line was used in this study.

Animals and other research organisms

Policy information about studies involving animals; ARRIVE guidelines recommended for reporting animal research, and Sex and Gender in Research

Laboratory animals

The female Atp6ap2flox/X mice (kindly provided by Dr. Frederique Yiannikouris, University of Kentucky; and Dr. Genevieve Nguyen, INSERM, France) were crossed with male osteocalcin (OCN)-Cre (kindly provided by Dr. Tom Clemens, Johns Hopkins Medical School) or DMP1-Cre (Jackson lab, Stock No 023047) transgenic mice to generate OB- or Osteocyte- selective conditional knockout (CKO) mutant mice, Atp6ap2OCN-Cre or Atp6ap2DMP1-Cre, respectively. Since Atp6ap2 is X-linked, only male mice were used in this study to ensure a sufficient number of littermates. The mutant mice were in C57BL/6J mouse background (for more than 6 generations). Unless otherwise indicated, male mice ranging from postnatal day 2 (P2) to postnatal day 90 (P90) were used in this study. The Ai9 and Axin2LacZ mice were purchased from the Jackson Laboratory (Stock No 007909 and 009120), which are in C57BL/6J background. All mice were maintained under specific pathogen-free (SPF) conditions in accordance with IACUC guidelines.

Wild animals

N/A

Reporting on sex

Since Atp6ap2 is X-linked, only male mice were used in this study to ensure a sufficient number of littermates.

Field-collected samples

N/A

Ethics oversight

All experimental procedures were approved by the Institutional Animal Care and Use Committee at Case Western Reserve University (IACUC, 2017-0121 and 2017-0115), according to US National Institutes of Health guidelines. We have complied with all relevant ethical regulations for animal use.

Note that full information on the approval of the study protocol must also be provided in the manuscript.

Plants

Seed stocks	N/A
Novel plant genotypes	N/A
Authentication	N/A
