# Supplementary material for: ATP6AP2 in osteoblasts couples cortical bone matrix remodeling to osteocyte maturation via MMP14 activation in mice
Source: Commun Biol. 2026 Jul 11;9:1454. doi: 10.1038/s42003-026-10615-z (PMC13737131; doi:10.1038/s42003-026-10615-z)
Supplement: Supplementary file 4 [file 42003_2026_10615_MOESM5_ESM.pdf]

nature portfolio

Peer Review File

ATP6AP2 in osteoblasts couples cortical bone matrix remodeling to osteocyte maturation via MMP14 activation in mice

Corresponding Author: Dr Wen-Cheng Xiong

This file contains all reviewer reports in order by version, followed by all author rebuttals in order by version.

Version 0:

Reviewer comments:

Reviewer #1

(Remarks to the Author)
ATP6AP2 in osteoblasts couples 1 cortical bone matrix remodeling to osteocyte distribution and maturation via MMP14 activation, by Lei Xiong et al, is an interesting manuscript reporting an osteoblast-specific deletion of the gene ATP6AT2, an accessory protein of the V-ATPase complex, previously shown to be active in a number of signaling and differentiation pathways including wnt/beta catenin signaling, known to be active in bone differentiation, and as previously reported by the authors, via the wnt receptor LRP6 in the osteoblast cell surface.
Mice were made by crossing floxed ATP6AP2 and osteocalcin-Cre mice, producing mice without ATPA6AP2 in osteoblast precursors, osteoblasts, and osteocytes. The key finding, not expected from related work on the gene, was that in 3 month old amimals, cortical bone volumes/total volumes and cortical thickness were increased but that the cortical bone was not compact an had low mineral density, pointing to a defect in bone quality. This and related morphology were consistent with greatly increased immatuere "woven bone" and poor osteocyte survival. Exhaustive and careful supporting work was done, showing that the bone is consistent with unremodeled "woven bone". This very interesting finding was followed by studying animals expressing tdTomato+ in mutant cortical bones, where the marker was not evenly distributed, in contrast to normal mice with OCNcre but not the ATP6AP2 deletion, where normal bone cells were labeled normally.
Beyond this, cell surface localization and activity were lost in vitro and in vivo in the conditional knockout, and detailed studies showed that ATP6AP2 interacts with MMP14 for surface targeting. Following this, expression of MMP14 in ATP6AP2-KO osteocytes restored collagen-degradative activity and rescued osteocyte maturation in ATP6AP2OCN-Cre96 animals. As with initial findings, work is reported in detail and included that the mutant mice showed disordered tdTomato relative to controls, with increased labeling in periosteum, the major site of cortical bone growth. There were major defects in osteocytic processes, which connect osteocytes. Defects were concentrated in SOST osteocytes, a maturity marker. Mice with the knockout concentrated in mature osteocytes using DMP1 cre were similar but with a weaker phenotype. Proteomic analysis and mRNA were studied, showing that ATP6AP2 probably regulates MMP14 protein trafficking and stability with expected effects on Type I collagen. MMP14 distribution showed an abnormal vesicular pattern. Further studies confirmed ATP6AP2 function in maintaining MMP14. ATP6AP2 interaction with MMP14 via its ICD/M8.9 site was shown by co-immunoprecipitation. Further, lentivirus-MMP14-eGFP in mutant OBs diminished the deficit. Detailed studies of cortical osteocyte properties were also done with consistent results.
A comprehensive but admirably brief Discussion is provided.

Comments:
Please indicate ages of animals directly in days.
Antibody labeling is not staining; immunofluorescence labeling is preferred, not staining.
Please spell out Ocys (osteoblastic osteocytes).

Reviewer #2

(Remarks to the Author)
Summary
This manuscript investigates the role of ATP6AP2 in osteoblast-lineage cells in regulating cortical bone development, with a focus on osteocyte differentiation, distribution, and matrix remodelling. Using conditional knockout mouse models, the

authors report that loss of ATP6AP2 leads to increased but structurally abnormal cortical bone characterised by retained woven bone, disrupted osteocyte maturation, and altered cell distribution. Mechanistically, they identify MMP14 as a downstream effector whose trafficking and surface localisation are regulated by ATP6AP2 and demonstrate partial phenotypic rescue via MMP14 re-expression.
Overall, this is a strong and potentially impactful study addressing an important and underexplored aspect of bone biology, namely the coupling between osteoblast activity, osteocyte maturation, and matrix remodelling in cortical bone. The identification of an ATP6AP2–MMP14 axis is novel and of broad interest.

In my opinion, there are some areas that the authors should address to improve the manuscript.

1. The manscript begins (in the abstract and the introduction e.g., “Cortical bone differs structurally and cellularly from trabecular bone, containing osteoblast (OB)-lineage–derived osteocytes”. Focussing on the difference between trabecular and cortical bone. Given that trabecular bone is not described in this manuscript, and that both bone types contain osteocytes, this undermines the narrative. I recommend removing the reference to trabecular bone in both instances and instead focussing on the gaps in knowledge regarding cortical bone specifically.

2. The manuscript repeatedly frames the increased bone mass seen in the knockout mice as “increased cortical bone formation”, however, the data only indicate increased thickness, increased porosity and woven bone retention. These findings are more consistent with defective maturation/remodelling, rather than necessarily increased formation. To address this directly, the authors could perform dynamic histomorphometry using double calcein labelling of bone as it is formed in their mutant mice. If this is not possible, they could reframe the findings as “accumulation of immature bone” and “defective lamellar bone maturation” and add as a limitation that they were not able to formally demonstrate bone formation and resorption rates.

3. The manuscript demonstrates differences in quantity of MMP14 and to some degree accesses its localisation in the different mutant mice but does not address its activity. This could be resolved through zymography rather than western blotting.

4. The authors introduce their previous work on ATP6AP2 by discussing its role in Wnt signalling. But effects on Wnt are not described in this manuscript. Are there differences in Wnt signalling in the mutant mice osteocytes?

5. Can the authors perform mechanical testing (e.g., 3-point bending) to assess the effect of the increased woven bone on structural integrity in the KO mice?

Minor errors identified:

1. Please use the full name of the gene the first time it appears in the abstract and introduction.

2. End of introduction: “Consistent with these findings, we recently reported that ATP6AP2 interacts with LRP6 in osteoblastic cells, where it promotes LRP6 cell surface localization and sustains both basal and Wnt-induced -βcatenin signaling, thereby regulating OB differentiation and trabecular bone formation. Notably, ATP6AP2-dependent Wnt/β-catenin signaling is not restricted to OBs but is also present in osteoclasts. Moreover, this regulatory mechanism appears to be specific to trabecula bone, with little or no effect observed in cortical bones. These observations raise an important unresolved question: does ATP6AP2 in osteoblasts contribute to cortical bone remodeling and homeostasis, and if so, through what mechanisms?”
Typo = trabecula
Given that this section states that there is “little or no effect observed in cortical bones” the logic that there are unresolved questions about how ATP6AP2 functions in cortical bone remodelling doesn’t function. Please revise and better explain the link and the previous findings.

3. End of page 4: “Re-expression of MMP14” is overly simplistic. Please state the types of targeting strategies used.

4. Scale bars are required for insert histology images e.g., figure 1, part F and figure 2 parts A and C.

5. No loading control is given for the membrane fraction in the western blot in figure 5.

6. Methods – references or method of genetic modification are required for each of the mice.

7. Methods – give additional information regarding housing and feeding of mice.

Reviewer #3

(Remarks to the Author)
The manuscript presents a detailed analysis of the consequences of deletion of ATP6AP2 in the osteoblastic lineage under OCN promoter. The authors demonstrate significant alterations in cortical bone, which they attribute primarily to impaired osteocyte differentiation caused by reduced MMP14 surface expression. The study is conceptually complementary to their previous work showing that trabecular bone defects arise from impaired LRP6-dependent Wnt/β-catenin signaling.
The findings are novel, mechanistically interesting, and potentially important for understanding cortical bone biology.

However, several conceptual and methodological issues require clarification or additional data before the manuscript can be considered for publication.
Mayor comments:
1) Although the authors propose a clear functional separation—LRP6/Wnt impairment in trabecular bone versus MMP14 deficiency in cortical bone—the data presented do not fully support a strict dichotomy. The phenotypes may overlap, and the contribution of Wnt/β-catenin signaling to cortical bone defects remains insufficiently addressed.
2) The authors report decreased ALP levels and impaired mineralization in cortical bone. It is unclear whether these changes arise solely from MMP14 deficiency or whether they are partially linked to altered Wnt/β-catenin signaling, as ALP is a classical target downstream of Wnt in osteoblast differentiation. A direct evaluation of the Wnt/β-catenin pathway in the ATP6AP2^OCN-Cre cortical compartment is essential to properly interpret these findings.
3)Given the critical importance of ATP6AP2 as a regulator of the Wnt pathway, a clear assessment of canonical Wnt activity in the cortex is necessary. Without this information, it remains speculative to assign cortical phenotypes exclusively to the ATP6AP2–MMP14 axis. A complete characterization of Wnt pathway status is fundamental to contextualize the cortical phenotype.
4)The rescue experiments are compelling, but the presentation can be significantly strengthened.
To properly interpret the extent of rescue, control animals should be included directly within Figure 7, allowing immediate comparison between wild-type, KO, and rescued conditions.
5)The manuscript does not address osteoclast activity in the ATP6AP2^OCN-Cre model. This is relevant as the woven-to-lamellar transition requires coordinated osteoclastic and osteoblastic activity.
Are osteoclasts functional in the KO model? Does MMP14 re-expression restore osteoclast behavior?
Could the cortical phenotype arise partly from defects in pathways unrelated to MMP14?
Minor comment:
In Figure S5C, the graph indicates that a CHX concentration of 100 µg/ml was used, whereas the figure legend states 50 µg/ml. Which concentration is correct?

Version 1:

Reviewer comments:

Reviewer #2

(Remarks to the Author)
I confirm that the reviewers have sufficiently addressed my comments in their rebuttal

Reviewer #3

(Remarks to the Author)
I appreciate the authors for the changes made.

Open Access This Peer Review File is licensed under a Creative Commons Attribution 4.0 International License, which permits use, sharing, adaptation, distribution and reproduction in any medium or format, as long as you give appropriate

credit to the original author(s) and the source, provide a link to the Creative Commons license, and indicate if changes were made.
In cases where reviewers are anonymous, credit should be given to 'Anonymous Referee' and the source.
The images or other third party material in this Peer Review File are included in the article's Creative Commons license, unless indicated otherwise in a credit line to the material. If material is not included in the article's Creative Commons license and your intended use is not permitted by statutory regulation or exceeds the permitted use, you will need to obtain permission directly from the copyright holder.
To view a copy of this license, visit https://creativecommons.org/licenses/by/4.0/

Reviewer #1:

"*ATP6AP2 in osteoblasts couples cortical bone matrix remodeling to osteocyte distribution and maturation via MMP14 activation, by Lei Xiong et al, is an interesting manuscript reporting an osteoblast-specific deletion of the gene ATP6AT2, an accessory protein of the V-ATPase complex, previously shown to be active in a number of signaling and differentiation pathways including wnt/beta catenin signaling, known to be active in bone differentiation, and as previously reported by the authors, via the wnt receptor LRP6 in the osteoblast cell surface*." "*Mice were made by crossing floxed ATP6AP2 and osteocalcin-Cre mice, producing mice without ATPA6AP2 in osteoblast precursors, osteoblasts, and osteocytes. The key finding, not expected from related work on the gene, was that in 3 month old amimals, cortical bone volumes/total volumes and cortical thickness were increased but that the cortical bone was not compact an had low mineral density, pointing to a defect in bone quality. This and related morphology were consistent with greatly increased immatuere "woven bone" and poor osteocyte survival. Exhaustive and careful supporting work was done, showing that the bone is consistent with unremodeled "woven bone". This very interesting finding was followed by studying animals expressing tdTomato+ in mutant cortical bones, where the marker was not evenly distributed, in contrast to normal mice with OCNcre but not the ATP6AP2 deletion, where normal bone cells were labeled normally.*"

"*Beyond this, cell surface localization and activity were lost in vitro and in vivo in the conditional knockout, and detailed studies showed that ATP6AP2 interacts with MMP14 for surface targeting. Following this, expression of MMP14 in ATP6AP2-KO osteocytes restored collagen-degradative activity and rescued osteocyte maturation in ATP6AP2OCN-Cre96 animals. As with initial findings, work is reported in detail and included that the mutant mice showed disordered tdTomato relative to controls, with increased labeling in periosteum, the major site of cortical bone growth. There were major defects in osteocytic processes, which connect osteocytes. Defects were concentrated in SOST osteocytes, a maturity marker. Mice with the knockout concentrated in mature osteocytes using DMP1 cre were similar but with a weaker phenotype. Proteomic analysis and mRNA were studied, showing that ATP6AP2 probably regulates MMP14 protein trafficking and stability with expected effects on Type I collagen. MMP14 distribution showed an abnormal vesicular pattern. Further studies confirmed ATP6AP2 function in maintaining MMP14. ATP6AP2 interaction with MMP14 via its ICD/M8.9 site was shown by co-immunoprecipitation. Further, lentivirus-MMP14-eGFP in mutant OBs diminished the deficit. Detailed studies of cortical osteocyte properties were also done with consistent results. A comprehensive but admirably brief Discussion is provided.*"

Response: We thank the reviewer for above positive and constructive comments. We are pleased that the reviewer finds our study interesting and comprehensive. We have carefully revised the manuscript according to the suggestions, as detailed below.

1."Please indicate ages of animals directly in days".

Response: Done. The ages of animals in days have been indicated in the revised manuscript throughout the text and Figures.

2. "Antibody labeling is not staining; immunofluorescence labeling is preferred, not staining".

Response: Agree! The term "immunofluorescence staining" has been replaced with "immunofluorescence labeling" throughout the manuscript at appropriated places.

3. "Please spell out Ocys (osteoblastic osteocytes)".

Response: Yes, we have now spelled out "Ocys" as "Osteocytes" in the manuscript.

Reviewer #2:

"This manuscript investigates the role of ATP6AP2 in osteoblast-lineage cells in regulating cortical bone development, with a focus on osteocyte differentiation, distribution, and matrix remodelling. Using conditional knockout mouse models, the authors report that loss of ATP6AP2 leads to increased but structurally abnormal cortical bone characterised by retained woven bone, disrupted osteocyte maturation, and altered cell distribution. Mechanistically, they identify MMP14 as a downstream effector whose trafficking and surface localisation are regulated by ATP6AP2 and demonstrate partial phenotypic rescue via MMP14 re-expression.
Overall, this is a strong and potentially impactful study addressing an important and underexplored aspect of bone biology, namely the coupling between osteoblast activity, osteocyte maturation, and matrix remodelling in cortical bone. The identification of an ATP6AP2–MMP14 axis is novel and of broad interest".

Response: We thank the reviewer for the thorough and insightful evaluation of our manuscript. We are encouraged by the positive assessment of the novelty and significance of our findings. We have carefully revised the manuscript to address all concerns, including clarifying the narrative, refining our interpretations, and providing additional methodological details. Our point-by-point responses are provided below.

1. "*The manscript begins (in the abstract and the introduction e.g., "Cortical bone differs structurally and cellularly from trabecular bone, containing osteoblast (OB)-lineage–derived osteocytes". Focussing on the difference between trabecular and cortical bone. Given that trabecular bone is not described in this manuscript, and that both bone types contain osteocytes, this undermines the narrative. I recommend removing the reference to trabecular bone in both instances and instead focussing on the gaps in knowledge regarding cortical bone specifically*".

Response: We thank the reviewer for this thoughtful comment. We agree that the primary focus of this study is cortical bone, and we have revised the Abstract and Introduction to reduce overemphasis on direct comparisons with trabecular bone. However, we have retained brief references to trabecular bone where necessary to provide context for previously established roles of ATP6AP2 in osteoblast differentiation and bone formation. These references are important for highlighting the current knowledge gap regarding its function in cortical bone. We have clarified this distinction in the revised text to ensure that trabecular bone is presented only as contextual background rather than a parallel focus of the study (see pages 2-5).

2. *"The manuscript repeatedly frames the increased bone mass seen in the knockout mice as "increased cortical bone formation", however, the data only indicate increased thickness, increased porosity and woven bone retention. These findings are more consistent with defective maturation/remodelling, rather than necessarily increased formation. To address this directly, the authors could perform dynamic histomorphometry using double calcein labelling of bone as it is formed in their mutant mice. If this is not possible, they could reframe the findings as "accumulation of immature bone" and "defective lamellar bone maturation" and add as a limitation that they were not able to formally demonstrate bone formation and resorption rates".*

Response: We thank the reviewer for this insightful comment. We agree that the phenotypes

observed in our current study—including increased cortical thickness, porosity, and retention of woven bone—are consistent with impaired bone maturation and defective remodeling. In line with this interpretation, we have revised the manuscript to more cautiously describe these changes as 'accumulation of immature bone' and 'defective lamellar bone maturation,' and we acknowledge that these features may reflect altered remodeling dynamics rather than simply increased bone formation.

In addition, we have carried out the suggested bone formation assay using calcein double-labeling. The new data are now included in the revised Figure 3k–n, which show an increased bone formation rate in the cortical bones of ATP6AP2 mutant mice, suggesting that elevated bone formation may also contribute to the increased cortical bone thickness. These results and points are described in the revised manuscript (see pages 7 and 16 in both the Results and Discussion sections)."

3. "*The manuscript demonstrates differences in quantity of MMP14 and to some degree accesses its localisation in the different mutant mice but does not address its activity. This could be resolved through zymography rather than western blotting*".

Response: We thank the reviewer for this valuable suggestion and agree that direct assessment of MMP14 enzymatic activity (e.g., by zymography) would further strengthen the study. However, it is important to note that gelatin zymography primarily detects soluble gelatinases such as MMP2 and MMP9 and is not well suited for evaluating the activity of membrane-anchored proteases like MMP14.
In the current study, we therefore measured collagen degradation capacity, a well-established surrogate of MMP14 function in type I collagen turnover. Our results demonstrate that collagen-degradative activity is significantly impaired in ATP6AP2-deficient cells (see revised Fig. 5k, l) and can be rescued by MMP14 expression (see Fig. S8d, e). These findings support the conclusion that MMP14 activity is impaired in ATP6AP2-deficient cells. We have included these points in the revised manuscript (see page 12 and 14)."

4. "The authors introduce their previous work on ATP6AP2 by discussing its role in Wnt signalling. But effects on Wnt are not described in this manuscript. Are there differences in Wnt signalling in the mutant mice osteocytes?"

Response: We thank the reviewer for this important comment. In the revised manuscript, we have directly addressed the role of the Wnt/β-catenin signaling pathway in our mouse model through additional in vivo analyses.

Using immunofluorescence labeling for active β-catenin (non-phospho Ser33/37/Thr41), we found that ATP6AP2 deletion led to a marked reduction of β-catenin in osteoblasts within the trabecular bone region, consistent with previous reports. In cortical bone, ATP6AP2 deficiency resulted in reduced β-catenin activity in periosteal cells, whereas an unexpected increase was observed within cortical bone matrix cells, particularly in enlarged tdTom-positive osteocytes (see revised Fig. S4a–c). These results suggest spatially distinct regulation of Wnt/β-catenin signaling by ATP6AP2 in osteoblast-lineage cells. We further validated this observation using $ATP6AP2^{OCN\text{-}Cre}$; $Axin2^{LacZ}$ reporter mice, which allowed us to assess Wnt/β-catenin activity via X-gal staining. Consistently, strong β-galactosidase activity was detected in the trabecular bone of control mice, and this activity was significantly reduced in ATP6AP2-deficient mice (see revised Fig. S4d–f). However, only minimal LacZ activity was observed in the cortical diaphysis of both control and mutant mice, with no obvious difference between groups (see revised Fig. S4d–f).

Together, these results demonstrate that ATP6AP2 is required for optimal Wnt/β-catenin signaling in osteoblasts of trabecular bone, whereas Wnt signaling activity in cortical osteocytes is low at baseline and appears less responsive to ATP6AP2 deletion under physiological conditions. We have revised the manuscript to include these new data and to clarify the compartment-specific effects of ATP6AP2 on Wnt/β-catenin signaling (see page 10).

5. "Can the authors perform mechanical testing (e.g., 3-point bending) to assess the effect of the increased woven bone on structural integrity in the KO mice?"

Response: We thank the reviewer for this valuable suggestion. Mechanical testing, such as three-point bending, would indeed provide important insight into the functional consequences of the structural abnormalities observed in the mutant mice.
However, we hope the reviewer will agree that such analyses were beyond the scope of the current study, which focused primarily on the cellular and molecular mechanisms underlying osteocyte maturation and matrix remodeling. We plan to address this important question in future investigations through collaboration with experts in biomechanical testing.

Minor errors identified:

1. "Please use the full name of the gene the first time it appears in the abstract and introduction".
Yes, revised.

2. "*End of introduction: "Consistent with these findings, we recently reported that ATP6AP2 interacts with LRP6 in osteoblastic cells, where it promotes LRP6 cell surface localization and sustains both basal and Wnt-induced -βcatenin signaling, thereby regulating OB differentiation and trabecular bone formation. Notably, ATP6AP2-dependent Wnt/β-catenin signaling is not restricted to OBs but is also present in osteoclasts. Moreover, this regulatory mechanism appears to be specific to trabecula bone, with little or no effect observed in cortical bones. These observations raise an important unresolved question: does ATP6AP2 in osteoblasts contribute to cortical bone remodeling and homeostasis, and if so, through what mechanisms?" Typo = trabecula. Given that this section states that there is "little or no effect observed in cortical bones" the logic that there are unresolved questions about how ATP6AP2 functions in cortical bone remodelling doesn't function. Please revise and better explain the link and the previous findings*".
We thank the reviewer for identifying these errors. We have corrected the typographical error and substantially revised this section to clarify the relationship between our previous findings and the current study. Importantly, we have removed the statement suggesting little or no effect of ATP6AP2 in cortical bone and revised the text to more accurately reflect that its role in cortical bone remains insufficiently characterized. These changes better justify the rationale for investigating ATP6AP2 in cortical bone and improve the logical flow of the Introduction (see page 4).

3. "End of page 4: "Re-expression of MMP14" is overly simplistic. Please state the types of targeting strategies used".
Yes, the detailed description of 'MMP14 expression' has been included in the revised manuscript (see page 5 and 14). Specifically, exogenous MMP14 was expressed using a Cre-dependent lentiviral system, enabling targeted overexpression specifically in OCN-Cre+ osteoblast-lineage cells within cortical bone by injection. This approach ensures cell-type–specific restoration of MMP14 expression in the conditional knockout mice.

4. "*Scale bars are required for insert histology images e.g., figure 1, part F and figure 2 parts A*

and C".
The Scale bars have now been added to all relevant histological images.

5. "No loading control is given for the membrane fraction in the western blot in figure 5".
We have now included the appropriate loading control (Neogenin) for the membrane fraction in Figure 5.

6. "Methods – references or method of genetic modification are required for each of the mice".
We have added appropriate references and detailed descriptions of the genetic models used.

7. "Methods – give additional information regarding housing and feeding of mice".
Okay. Additional details regarding animal housing and feeding conditions have been included in the Methods section.

Reviewer #3:

"*The manuscript presents a detailed analysis of the consequences of deletion of ATP6AP2 in the osteoblastic lineage under OCN promoter. The authors demonstrate significant alterations in cortical bone, which they attribute primarily to impaired osteocyte differentiation caused by reduced MMP14 surface expression. The study is conceptually complementary to their previous work showing that trabecular bone defects arise from impaired LRP6-dependent Wnt/β-catenin signaling.*
The findings are novel, mechanistically interesting, and potentially important for understanding cortical bone biology. However, several conceptual and methodological issues require clarification or additional data before the manuscript can be considered for publication".

We thank the reviewer for the thoughtful and constructive evaluation of our work. We are encouraged by the recognition of the novelty and potential significance of our findings. We agree that further clarification is needed regarding the relationship between ATP6AP2, Wnt/β-catenin signaling, and MMP14-dependent mechanisms in cortical bone. We have revised the manuscript accordingly to clarify our interpretations, refine our conclusions, and provide additional explanations where appropriate.

Major comments:

1."Although the authors propose a clear functional separation—LRP6/Wnt impairment in trabecular bone versus MMP14 deficiency in cortical bone—the data presented do not fully support a strict dichotomy. The phenotypes may overlap, and the contribution of Wnt/β-catenin signaling to cortical bone defects remains insufficiently addressed".

Response: We thank the reviewer for this important comment, and agree that the distinction between trabecular and cortical bone phenotypes should not be interpreted as a strict functional dichotomy.

In the revised manuscript, we have softened this interpretation and now emphasized that ATP6AP2 likely regulates multiple pathways in osteoblast-lineage cells, including Wnt/β-catenin signaling pathway and MMP14-dependent matrix remodeling, which may act in a partially overlapping and context-dependent manner.

To directly address this point, we have now included immunofluorescence labeling for active β-catenin as well as Axin2^{LacZ} reporter analysis. These data demonstrate reduced Wnt/β-catenin signaling in trabecular osteoblasts, whereas cortical bone shows low baseline Wnt activity with region-specific changes, supporting a more nuanced, compartment-dependent regulation rather than a strict separation (see revised Fig. S4 and the manuscript-page 10).

2) "The authors report decreased ALP levels and impaired mineralization in cortical bone. It is unclear whether these changes arise solely from MMP14 deficiency or whether they are partially linked to altered Wnt/β-catenin signaling, as ALP is a classical target downstream of Wnt in osteoblast differentiation. A direct evaluation of the Wnt/β-catenin pathway in the ATP6AP2$^{OCN\text{-}Cre}$ cortical compartment is essential to properly interpret these findings".

Response: We thank the reviewer for this insightful comment. We agree that the cortical bone phenotype is unlikely to be explained solely by MMP14 deficiency. ALP is a well-established downstream target of Wnt/β-catenin signaling during osteoblast differentiation. Therefore, it is plausible that alterations in Wnt signaling may also contribute to the observed cortical bone phenotype in ATP6AP2$^{OCN\text{-}Cre}$ mice.

We have revised the manuscript to clarify that the cortical bone defects likely reflect combined effects of disrupted Wnt/β-catenin signaling and impaired MMP14-mediated extracellular matrix remodeling, rather than a single downstream pathway (see page 17).

3)"Given the critical importance of ATP6AP2 as a regulator of the Wnt pathway, a clear assessment of canonical Wnt activity in the cortex is necessary. Without this information, it remains speculative to assign cortical phenotypes exclusively to the ATP6AP2–MMP14 axis. A complete characterization of Wnt pathway status is fundamental to contextualize the cortical phenotype".

Response: We agree with the reviewer that it is important to clarify the role of canonical Wnt signaling in cortical bone. In the revised manuscript, we have evaluated Wnt/β-catenin pathway in ATP6AP2$^{OCN\text{-}Cre}$ mice using both active β-catenin immunostaining and Axin2LacZ reporter mice (see revised Fig. S4). These analyses revealed that cortical bone exhibits low baseline Wnt/β-catenin activity under physiological conditions, with no global reduction following ATP6AP2 deletion; instead, reduced activity was observed specifically in trabecular osteoblasts (see revised Fig. S4). These findings indicate that the cortical bone phenotypes are unlikely to be driven exclusively by changes in canonical Wnt signaling. Rather, they support a model in which ATP6AP2 regulates cortical bone remodeling through integrated effects on both Wnt signaling and matrix remodeling pathways. These points have now been included in revised manuscript (see page 16-17).

4) "The rescue experiments are compelling, but the presentation can be significantly strengthened. To properly interpret the extent of rescue, control animals should be included directly within Figure 7, allowing immediate comparison between wild-type, KO, and rescued conditions".

Response: Yes, we agree with the reviewer that including control groups alongside knockout and rescue conditions improves clarity and interpretability. Accordingly, we have revised Figure 7 to include wild-type, knockout, and rescue groups within the same panel for direct comparison (see revised Fig. 7).

5) "The manuscript does not address osteoclast activity in the ATP6AP2^OCN-Cre model. This is relevant as the woven-to-lamellar transition requires coordinated osteoclastic and osteoblastic

activity.
Are osteoclasts functional in the KO model? Does MMP14 re-expression restore osteoclast behavior?
Could the cortical phenotype arise partly from defects in pathways unrelated to MMP14?"

Response: We thank the reviewer again for this important and insightful comment. We agree that coordinated osteoblastic and osteoclastic activities are essential for proper bone remodeling and the trabecular-to-lamellar transition. In the revised manuscript, we have addressed this question and obtained the following findings:

1. Increased osteoclast activity in ATP6AP2$^{OCN\text{-}Cre}$ mice

TRAP staining and serum PYD measurements revealed increased osteoclast activity in the mutant mice (see revised Fig. S5a–c). To test the functional relevance of this increase, we treated mice with alendronate to inhibit osteoclast activity. Osteoclast inhibition significantly increased trabecular bone mass in both control and mutant mice; however, cortical bone defects in ATP6AP2-deficient mice were not rescued, indicating that cortical abnormalities are largely independent of osteoclast activity (see revised Fig. S5d–i).

2. Do osteoclasts normalize after MMP14 expression?

We did not directly assess osteoclast number or activity following MMP14 re-expression, and whether MMP14 restoration has a direct impact on osteoclast function remains to be determined in future studies. However, given that osteoclast activity is increased rather than reduced in ATP6AP2$^{OCN\text{-}Cre}$ mice (see revised Fig. S5d–i) and that osteoclast inhibition primarily affects trabecular but not cortical bone phenotypes (revised Fig. S5), we speculate that the rescue effects of MMP14 expression act primarily through osteoblast-lineage–mediated matrix remodeling and osteocyte maturation, rather than via direct regulation of osteoclasts. We have revised the manuscript to state this limitation and to avoid overinterpretation regarding potential effects of MMP14 on osteoclast biology (see page 11).

3. Could cortical phenotypes arise from MMP14-independent pathways?

We agree with the reviewer that the cortical phenotype cannot be fully explained by MMP14 alone. Our osteoclast analyses further support this interpretation, as cortical bone defects persist despite effective suppression of osteoclast activity by alendronate treatment. This indicates that cortical abnormalities are largely osteoclast-independent and likely arise from additional pathways regulated by ATP6AP2, including matrix remodeling and potentially other signaling mechanisms beyond MMP14.

In summary:

- Osteoclast activity is increased, not reduced, in ATP6AP2-deficient mice.
- Trabecular bone loss is partially osteoclast-dependent.
- Cortical bone defects are largely osteoclast-independent.
- MMP14 primarily regulates osteoblast-lineage matrix remodeling rather than osteoclast function.

We have revised the manuscript accordingly to reflect these findings and to clarify the compartment-specific mechanisms underlying the observed skeletal phenotypes (see page 11).

Minor comment:

"In Figure S5C, the graph indicates that a CHX concentration of 100 µg/ml was used, whereas the figure legend states 50 µg/ml. Which concentration is correct?"

The concentration is 50 µg/ml, which has been revised.
